# A systematic review of immersive educational technologies in medical physics and radiation physics

**DOI:** 10.3389/fmed.2024.1384799

**Published:** 2024-10-10

**Authors:** Talia Tene, Nataly Bonilla García, Diana Coello-Fiallos, Myrian Borja, Cristian Vacacela Gomez

**Affiliations:** ^1^Department of Chemistry, Universidad Técnica Particular de Loja, Loja, Ecuador; ^2^Facultad de Ciencias, Escuela Superior Politécnica de Chimborazo (ESPOCH), Riobamba, Ecuador; ^3^Grupo de Investigación Ciencia de Datos (CIDED), Escuela Superior Politécnica de Chimborazo (ESPOCH), Riobamba, Ecuador; ^4^INFN-Laboratori Nazionali di Frascati, Frascati, Italy

**Keywords:** immersive technologies, virtual reality, augmented reality, mixed reality, medical physics, radiation physics, PICOS approach, PRISMA framework

## Abstract

**Objective:**

This systematic review aims to analyze and synthesize the current state of research on the role of immersive technologies, specifically augmented reality (AR), virtual reality (VR), and mixed reality (MR), in medical physics and radiation physics education. The primary focus is to evaluate their impact on learning outcomes, performance, and engagement across various educational contexts.

**Methods:**

We conduct a comprehensive search of four major databases: Scopus, Web of Science, PubMed, and IEEE Xplore, covering the period from 2012 to 2023. A total of 316 articles are initially identified. After removing duplicates and screening for relevance based on titles and abstracts, 107 articles are selected for full-text review. Finally, 37 articles met the inclusion criteria and are included in the analysis. The review follows the PRISMA guidelines and utilizes the PICOS framework to structure the research question.

**Analysis:**

Data extraction focuses on key variables such as the type of immersive technology used, educational context, study design, participant demographics, and measured outcomes. The studies are analyzed for their reported effects on learning outcomes, performance, and engagement.

**Results:**

The review found that immersive technologies significantly enhance learning outcomes and engagement. Specifically, 36.4% of the studies reported increased engagement, while 63.6% of studies focusing on practical skills noted performance improvements. The use of AR, VR, and MR showed broad applicability across different educational levels, from undergraduate courses to professional training programs.

**Conclusion:**

Immersive technologies have considerable potential to transform medical and radiation physics. They enhance student engagement, improve learning outcomes, and boost performance in practical skills. Nevertheless, future research should focus on standardizing methodologies, expanding participant demographics, and exploring long-term impacts on skill retention and clinical practice. This review provides a valuable resource for guiding future research and implementing innovative educational strategies in the dynamic fields of medical physics and radiation physics.

## 1 Introduction

Immersive technologies, encompassing virtual reality (VR), augmented reality (AR), and mixed reality (MR), have emerged as transformative tools in various educational domains (1). These technologies create interactive and engaging learning experiences by simulating complete virtual environments, overlaying digital information in the real world, or integrating real and virtual elements for real-time interaction (2). The adoption of these technologies in education is driven by their ability to provide hands-on, experiential learning opportunities that traditional methods often lack (3).

In the specialized fields of medical physics and radiation physics, precise knowledge and practical skills are essential. Medical physics involves the application of physics principles to medicine, particularly in the diagnosis and treatment of diseases. This field encompasses areas such as medical imaging [e.g., X-rays (4)], radiation therapy (5), nuclear medicine (6), and health physics (7). Indeed, medical physicists work to develop, optimize, and ensure the safety of these technologies, contributing to advancements in diagnostic accuracy and therapeutic effectiveness. On the other hand, radiation physics, a subfield of medical physics, focuses specifically on the study and application of ionizing radiation in medical treatments (8). One of the primary areas of radiation physics is cancer radiotherapy (9), where high-energy radiation is used to destroy cancer cells while minimizing damage to surrounding healthy tissues. This involves complex planning and precise delivery of radiation doses, requiring a deep understanding of both the physics of radiation and its biological effects on human tissues (10). Radiation physicists are integral in designing treatment plans, calibrating equipment, and ensuring that radiation doses are accurately delivered.

As stated, immersive technologies hold the potential to address the limitations of traditional training methods by offering realistic simulations and interactive learning environments (11). Then, in medical physics and radiation physics, VR can simulate complex clinical procedures and treatment planning (12), AR can enhance real-world training by overlaying anatomical or procedural information (13), and MR can provide an integrated learning experience that combines physical and virtual elements (14).

For instance, VR has been used to simulate radiation therapy procedures, allowing students to practice and understand the intricate process of targeting tumors while sparing healthy tissue (15). Additionally, VR is utilized in surgical training, where trainees can practice surgeries in a virtual environment, enhancing their skills without risking patient safety (16). AR overlays digital information onto the real world, enhancing the user’s perception and interaction with their environment. For example, AR applications based on the Microsoft HoloLens system, have been employed to project 3D models of anatomical structures onto patients, providing medical students and professionals with a deeper understanding of spatial relationships and procedural steps (17). Furthermore, AR has been used in radiology to overlay medical images onto a patient’s body, aiding in more precise localization and diagnosis (18). MR combines elements of both VR and AR, integrating real and virtual components for an interactive experience. An example of MR in education is the use of VR headsets in conjunction with physical mannequins in surgical training, offering a blended experience where learners can interact with both virtual and real elements (19). MR is also used in radiation therapy planning, where it helps in visualizing radiation dose distributions in a more intuitive way (20).

As noted, these immersive technologies can offer several opportunities and advantages over traditional training methods. Traditional methods in medical and radiation physics education typically involve theoretical instruction, laboratory exercises, and clinical practice (21). While these methods are foundational, they have certain limitations, such as limited access to advanced equipment and technology, safety concerns associated with realistic training, and the challenge of replicating complex clinical scenarios in a controlled setting. In contrast, immersive technologies can provide enhanced engagement through interactive and immersive experiences, creating a safe learning environment where students can practice procedures and make mistakes without risking patient safety (22). They also facilitate accessibility to advanced training scenarios that may be rare or resource-intensive in real life and improve the understanding of complex concepts through detailed visualizations and interactive elements (23).

Despite the promising potential of immersive technologies, the current research scenery in their application to medical physics and radiation physics education is still developing. Many studies focus on isolated applications or small-scale implementations, lacking a comprehensive overview of their impact on learning outcomes. Additionally, there is a need for systematic evaluations to compare the effectiveness of immersive technologies with traditional training methods and to identify best practices for their integration into curricula. To address these gaps, this systematic review aims to answer the following research question:

- “*How do immersive technologies contribute to medical physics and radiation physics education and training*?”

The primary objective of this systematic review is to evaluate the effectiveness of immersive technologies in enhancing education and training in medical physics and radiation physics. Specifically, we aim to identify the types of immersive technologies used in these fields, assess their impact on learning outcomes and skills development, highlight the benefits and limitations of using immersive technologies in education and training, and provide recommendations for best practices in their integration into educational curricula. By systematically analyzing the existing literature, this paper seeks to provide a comprehensive understanding of the current state of immersive technology use in medical and radiation physics education, identify gaps in the research, and suggest directions for future studies.

## 2 Methodology

Conducting a systematic review is crucial for synthesizing the wide array of literature. This methodical approach enables the identification, evaluation, and integration of findings from diverse studies, providing a comprehensive and unbiased summary of the current evidence. Systematic reviews help to clarify the effectiveness and potential benefits of immersive technologies, uncover gaps in the existing research, and offer evidence-based recommendations for future studies and practical applications.

Then, the goal of this systematic review is to analyze and synthesize the current state of research on the role of immersive technologies in medical physics and radiation physics education and training. While several narrative reviews are available on related topics (24, 25) (discussed in section 4), a systematic review is straightway needed to provide a more structured, transparent, and replicable assessment of the existing literature. The population, intervention, comparison, outcomes, and study design (PICOS) framework (26) is essential for structuring the research question to guide the systematic review process. By clearly defining these components, the PICOS approach ensures that the review addresses specific and relevant aspects of the research topic, thereby enhancing the focus and relevance of the findings. The components of the research question for this systematic review are outlined in Table 1.

- In terms of population, the review focuses on both students and professionals in the fields of medical physics and radiation physics. This includes undergraduate and graduate students, as well as practicing professionals who are undergoing continuous education and training. Understanding how immersive technologies impact different levels of learners and practitioners is crucial for evaluating their overall effectiveness and applicability in these fields.
- The interventions of interest are the use of immersive technologies (VR, AR, MR) in education and training. The review aimed to capture various applications of these technologies, such as virtual simulations of radiation therapy procedures, augmented anatomical models, and mixed-reality surgical training environments.
- Traditional educational methods or other forms of training served as the comparison tool. These traditional methods typically involve theoretical instruction through lectures and textbooks, hands-on laboratory exercises, and clinical practice. Comparing immersive technologies to these established methods provides insights into their relative advantages and potential areas for improvement.
- The primary outcomes of interest were learning outcomes, performance, and engagement. Learning outcomes pertain to the acquisition of knowledge and skills through training, while performance reflects the ability to apply this knowledge and these skills in practical contexts. Engagement, on the other hand, encompasses the learners’ level of interest, motivation, and participation throughout the training process. Evaluating these outcomes is crucial for understanding the effectiveness of immersive technologies in enriching educational experiences and fostering meaningful learning.
- This inclusive approach allowed for a comprehensive understanding of the current state of research and the diverse methodologies employed to study the impact of immersive technologies.

**Table 1:** Components of the research question using the PICOS framework.

P	Population	Students and professionals in medical physics and radiation physics
I	Intervention	Use of immersive technologies (VR, AR, MR) in education and training
C	Comparison	Traditional educational methods or other forms of training
O	Outcomes	Learning outcomes, performance, and engagement
S	Study design	All study designs, including full articles, review articles, and conference papers

Additionally, the preferred reporting items for systematic reviews and meta-analyses (PRISMA) guidelines (27) offer a standardized methodology for reporting systematic reviews and meta-analyses. These guidelines enhance the transparency and completeness of the review process by ensuring that all relevant aspects are thoroughly documented and reported. Key elements of the PRISMA guidelines include the detailed documentation of the search strategy, clear articulation of selection criteria, systematic data extraction, rigorous assessment of the risk of bias, and the synthesis of results.

**Figure 1:**
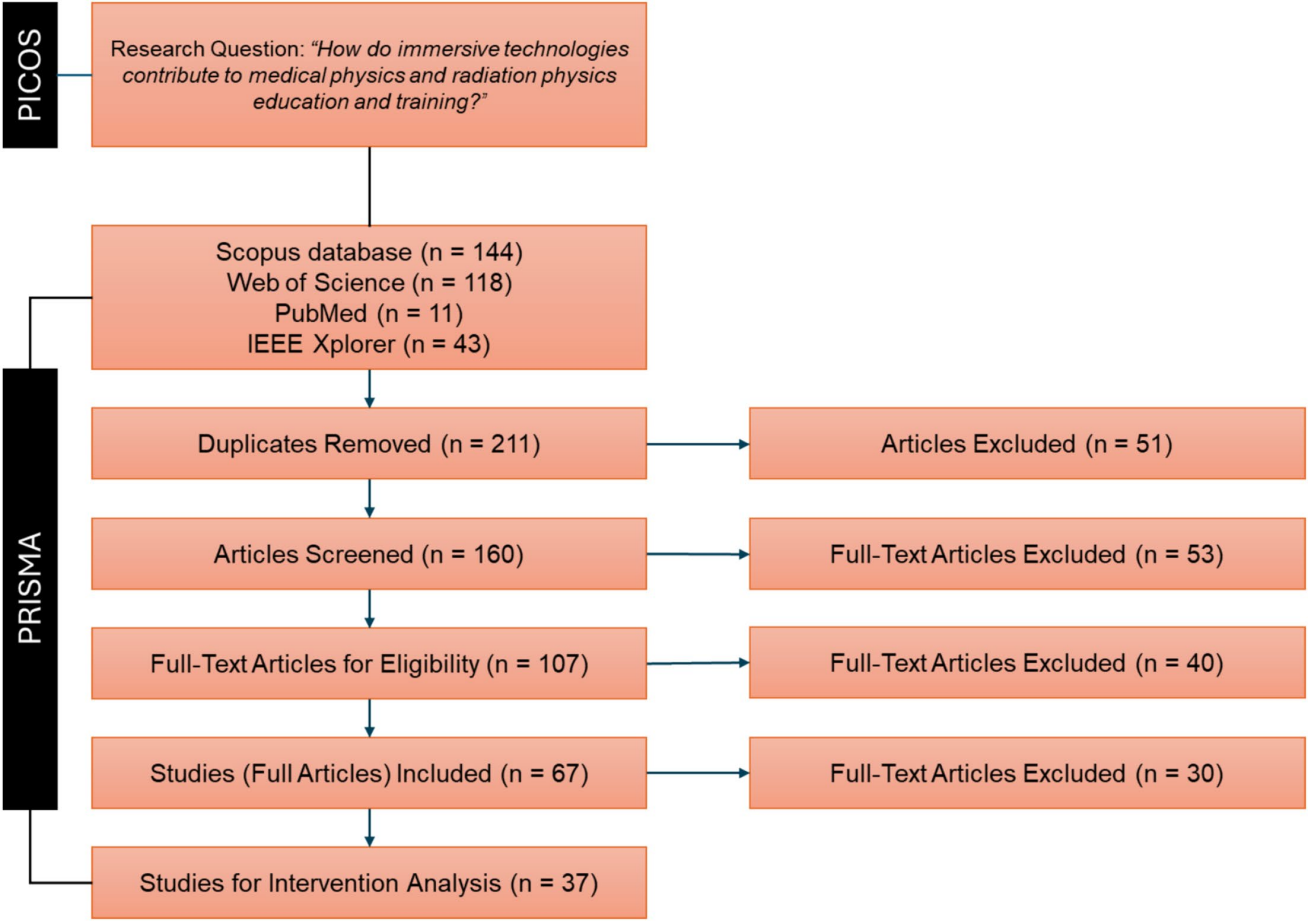
Flowchart of the systematic review process carried out in this work.

Combining the PRISMA guidelines with the PICOS framework ensures a robust and structured approach to our systematic review. This dual approach enables us to capture the latest advancements and trends in the use of immersive technologies in medical physics and radiation physics education, offering a comprehensive and up-to-date synthesis of the evidence. Figure 1 illustrates the systematic review process guided by these frameworks.

### 2.1 Identification stage

**Figure 2:**
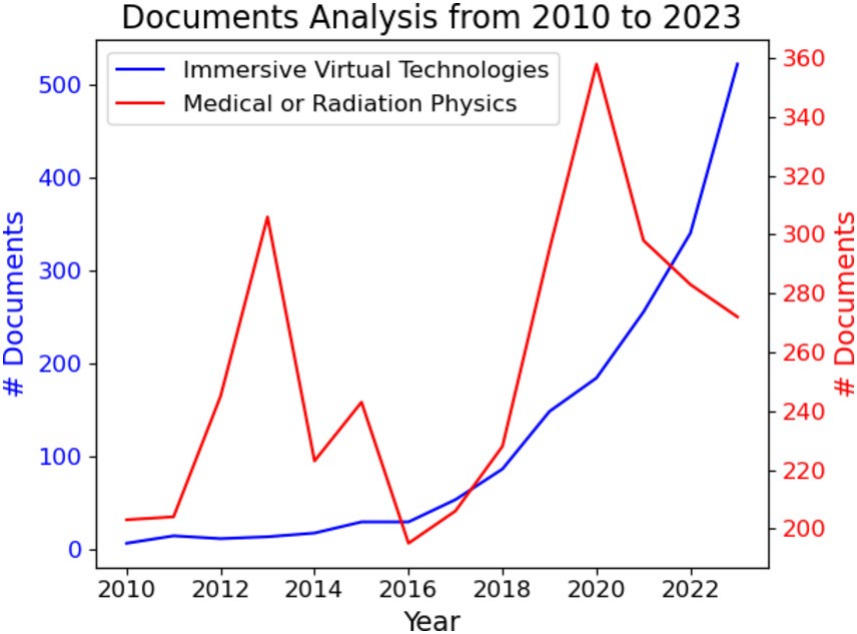
The number of documents identified by searching: “immersive virtual technology” (blue) and “integration of virtual technologies in medical or radiation physics” (red). Data was obtained from the Scopus database.

The chosen timeframe of 2012 to 2023 for this systematic review is crucial for several reasons:

- The field of immersive technologies has seen significant advancements and a surge in scientific publications over the past decade. The application of VR, AR, and MR in educational contexts, particularly in medical and radiation physics, has evolved substantially during this period. The increasing sophistication and accessibility of these technologies have led to numerous innovative applications in training and education (Figure 2).
- Starting in 2012, there was a noticeable increase in the number of studies focusing on the integration of immersive technologies in medical and radiation physics. This period captures the maturation of these technologies from basic research to more applied studies, including comparative studies that directly assess the performance improvements brought by immersive technologies (Figure 2).

On the other hand, the selection of Scopus, Web of Science, PubMed, and IEEE Xplore (see Figure 1 and Table 2) as the primary databases for this systematic review is strategic and justified by their comprehensive coverage, relevance, and reputation in the scientific community. These databases encompass a wide range of scientific disciplines and provide extensive coverage of journals and conference proceedings, making them essential resources for a systematic review (28–31). This strategic choice enhances the thoroughness and reliability of the review by capturing diverse perspectives and research findings related to the use of immersive technologies in education and training. Ultimately, in the identification stage of this systematic review, a precise and comprehensive query strategy was employed to retrieve relevant studies from the selected databases (see Table 2). The query was designed to encompass key terms related to the research topic. The query terms were carefully chosen to capture a wide range of studies while maintaining specificity to the topic.

**Table 2:** Query type and the corresponding results.

Database	Query	Results
Scopus	(“Immersive technologies” OR “Virtual reality” OR “Augmented reality”) AND (“Education” OR “Training” OR “Teaching”) AND (“Medical Physics” OR “Radiation Physics” OR “Physics” OR “Radiation” OR “Medicine”)	144
Web of Sciences		118
PubMed		11
IEEE Xplore		43

Year restriction applied from 2012 to 2023.

### 2.2 Screening stage

We initially identified 316 articles across the selected databases: 144 from Scopus, 118 from Web of Science, 11 from PubMed, and 43 from IEEE Xplore. After removing duplicates, 211 unique articles remained for further screening based on their titles and abstracts. The screening criteria were as follows:

- Inclusion of review articles, full research articles, and proceedings papers.
- Focus on articles specifically addressing immersive technologies.
- Relevance to medical physics and radiation physics education and training.
- Inclusion of articles irrespective of language.

During the screening process, 51 articles were excluded, resulting in 160 articles advancing to the next stage. The reasons for exclusion were:

- Thirty-eight articles did not focus on or include immersive technologies.
- Ten articles did not pertain to education or training in medical or radiation physics.
- Three articles were not available in full text.

### 2.3 Eligibility stage

During the eligibility phase, the articles were randomly assigned to the authors for a thorough full-text analysis. The eligibility criteria were as follows:

- The full text of the article is available in any language.
- The article focuses on the use of immersive technologies in education and training.
- The article centers on medical physics or radiation physics.
- The article specifically discusses the application of VR, AR, or MR in educational contexts.

At this stage, 53 articles were deemed ineligible, leaving 107 articles suitable for inclusion and data extraction. The reasons for exclusion were:

- Forty-one articles did not focus on educational applications of immersive technologies.
- Twelve articles were narrative review papers discussing the general topic of immersive technologies without a specific focus on medical or radiation physics education.

### 2.4 Included stage

To conclude the selection process, the eligible articles were thoroughly processed to extract all relevant interventions that impact the educational outcomes in medical physics and radiation physics training using immersive technologies. During this stage, 107 articles underwent comprehensive analysis, with each work evaluated based on the following key metrics:

- Learning outcomes.
- Performance.
- Engagement.

At this stage, 40 articles were deemed ineligible, resulting in 67 articles being considered suitable for further extraction and analysis. The reasons for exclusion were as follows:

- Forty articles did not report conclusive metrics. For example, some articles were excluded due to the lack of rigorous methodology or insufficient data on educational outcomes.

Additionally, out of these 67 articles, 30 were further excluded because they did not pertain exclusively to medical physics or radiation physics education in the context of application and evaluation. Consequently, 37 articles were selected for an in-depth study to contextualize and analyze the role of immersive technologies in enhancing education and training in these specialized fields.

## 3 Results and discussions

### 3.1 Summary of search results

To begin, 37 articles were included in the final data extraction. These articles were analyzed to extract parameters and interventions to contextualize the role of immersive technologies in medical physics and radiation physics education and training and their impact on various performance metrics. The interventions identified in the selected articles were categorized into several key aspects:

- Types of interventions include the specific immersive technology used (VR, AR, MR) and its application in educational contexts.
- Types of variables focus on performance and engagement metrics, evaluating how these technologies impact learning outcomes.
- Types of effects show the results as positive, negative, increased, decreased, or neutral, based on the observed impact on educational outcomes.
- Types of immersive technologies specify types of VR, AR, or MR technologies used in the studies.
- Stages of the immersive technologies are described as fully implemented, pilot stage, or prototype.
- Number of participants refers to the sample size of each study, providing context for the robustness of the findings.
- Study design represents the methodological design of each study, including randomized controlled trials, clinical studies, case studies, and qualitative research.
- Participants demographics refer to the information about the participants, such as their educational level, professional status, and other relevant demographics.
- Limitations and challenges identify key aspects in the implementation and evaluation of immersive technologies in educational settings.

### 3.2 Summary of interventions

The outcomes of the analyzed studies (32–68) can be found in Table 3 as well as expanded and further details in Supplementary Tables S1–S3. The evaluated studies display a variety of immersive technology interventions implemented in medical physics and radiation physics. Medical physics applies physics principles to medical imaging and treatments. Radiation physics, a subfield, focuses on using radiation like X-rays for diagnosis and therapy.

**Table 3:** Summary of Interventions considering variable, effect, and limitations of the work.

References	Intervention	Observed variable	Observed effect	Limitations
Rowe et al. (32)	VR simulation for radiography training and intracavitary brachytherapy using Virtual Medical Coaching’s software and CVVR and IHVR	Performance	Positive	Limited to first-year students, needs further research for complex procedures and other settings
Shah et al. (33)	VR simulation for intracavitary brachytherapy training using cardboard viewer VR (CVVR) and integrated headset VR (IHVR)	Learning outcomes	Positive	Single-institution study, small sample size, no 3D-video investigation, limited scope
Ryu et al. (34)	Mixed reality-based hologram for intraoperative navigation in colorectal surgery	Performance	Positive	Single patient case, outside study period
Pastor et al. (35)	Digitally enhanced hands-on surgical training (DEHST) for freehand distal interlocking of intramedullary nails	Performance	Neutral	Small number of participants, potential learning effect, task completion time excluded
Chen et al. (36)	VRContour, a VR-based tool for contouring medical structures	Performance	Positive	Small sample size, limited VR experience
Kiryukhin et al. (37)	Virtual analog of uranium-water subcritical assembly for education and training	Learning outcomes, engagement	Positive	Details on sample size and control group not provided
Bridge et al. (38)	Simulation-based education (SBE) including VR simulators, computer-based systems, and simulated patients	Learning outcomes, engagement	Positive	Details on sample size and control group not provided
Ma and Alghamdi (39)	Physical mannequin representing the patient with an online Monte Carlo simulation package generating synthetic images in real time	Learning outcomes, engagement	Positive	Small sample size, respondent bias, geographical limitations
Wang et al. (40)	Unguided trauma simulation practice using the TraumaVision VR Simulator (Swemac) for distal locking screw placement	Performance	Positive	Limited simulation of abnormalities, issues with breathing and motion artifacts
Gunn et al. (41)	VR CT simulation for medical imaging (MI) and radiation therapy (RT) undergraduate students to learn CT scanning	Learning outcomes, engagement	Positive	No significant improvement in radiation use or overall score
Martin-Gomez et al. (42)	AR system using HoloLens2 to provide visual feedback of patient’s respiratory trace during SBRT for pancreatic cancer treatment	Learning outcomes, engagement	Positive	Self-reported confidence, no pre/post knowledge skill test, changing course structure
Taunk et al. (43)	VR-based intracavitary brachytherapy simulation for gynecologic brachytherapy training	Learning outcomes	Positive	Preliminary study, small sample size, need for comprehensive patient study
Czaplinski and Fielding (44)	Blended learning framework with VERT (virtual environment radiotherapy training) simulations for medical physics students	Learning outcomes, engagement	Positive	Small sample size, single institution study
Nishi et al. (45)	AR application for visualizing the spread of scattered radiation in radiography using AR	Learning outcomes, engagement	Positive	Small sample size, low response rate, single institution study
Kang et al. (46)	Gross anatomy laboratory sessions with AR tools for medical physics students	Learning outcomes, engagement	Positive	Preliminary study, small sample size, need for comprehensive study
Johnson et al. (47)	360-degree VR video outlining the technical aspects of EBRT to the pelvis as a supplement to traditional education methods	Learning outcomes, engagement	Positive	Small sample size, single institution study
Park et al. (48)	3D AR visualization of preprocedural MR images for guiding transarterial embolization in a preclinical model of hepatocellular carcinoma	Performance	Positive	Small sample size, single institution study, English-speaking only, potential researcher bias
Sapkaroski et al. (49)	VR simulation using CETSOL VR Clinic software for radiographic positioning training versus traditional clinical role-play	Learning outcomes, engagement	Positive	Small sample size, single-center study, potential implicit bias
Jones et al. (50)	VR training simulator for cochlear implant surgery developed using Unity3D with haptic feedback for electrode insertion	Performance	Neutral	Small sample size, single institution study, limited to hand positioning training
Gu and Lee (51)	AR dental radiography simulator for preclinical training, allowing students to practice on a 3D manikin head using a mobile device with real-time feedback	Learning outcomes, engagement	Neutral	Preliminary study, small sample size, lack of detailed participant demographics
Ryan and Poole (52)	Virtual learning environment (VLE) for radiation therapy education	Learning outcomes, engagement	Positive	Preliminary study, small sample size, lack of detailed participant demographics, iOS only initially
Popovic et al. (53)	Simulation training in coronary angiography using the Simbionix Angio-Mentor	Performance	Positive	Small sample size, potential bias from single researcher
Fernández et al. (54)	Use of VR devices to simulate real-world conditions and measure radiation absorption in the brain and eyes of children compared to adults	Performance	Negative	Small sample size, single center, potential biases, difficulty isolating simulation effects
Guo et al. (55)	AR system for antenna design education using mobile phones, smart gloves, and virtual buttons	Engagement	Positive	Emphasizes need for refined regulatory testing and considers age-specific absorption rates
Sugand et al. (56)	AR simulator for hip surgery guide-wire insertion using Logitech cameras, a phantom limb, a rotary drill, and a guide-wire	Performance	Positive	More suitable for beginners, needs further development for professional use
Sapkaroski et al. (57)	CETSOL VR Clinic, a haptic feedback VR simulation for medical imaging students using Oculus Rift and HTC Vive for dynamic patient interaction and clinical exams	Learning outcomes, engagement	Positive	Did not record number of DHS procedures, potential time constraints influence, lack of hand dominance consideration
Gunn et al. (58)	VR simulation for training first-year medical imaging students in a virtual x-ray room with interactive equipment and patient	Learning outcomes, performance	Positive	Comparison limited to consecutive year groups, confined to hand positioning task
Chamunyonga et al. (59)	VERT system for teaching IMRT, VMAT, DCAT planning, and QA, evaluating dose coverage and OAR sparing with ArcCHECK and IMRT phantoms	Learning outcomes, engagement	Positive	Assessed only technical skills, did not evaluate satisfaction or enjoyment, needs further research for clinical outcomes and confidence
Diotte et al. (60)	AR fluoroscope for assisting surgeons in distal locking of intramedullary nails, integrating optical and X-ray images	Performance	Positive	Relies on anecdotal evidence and qualitative assessment, needs further quantitative research
Szőke et al. (61)	VRdose and Halden Planner for 3D radiation risk assessment and work simulation in nuclear environments	Performance	Positive	Conducted on dry bone phantoms, needs further research in clinical settings, small sample size
Nishi et al. (62)	Development and use of a WebAR system to visualize scattered radiation during portable imaging using the Monte Carlo method	Learning outcomes, engagement	Positive	Primarily descriptive, lacks quantitative measurement of educational outcomes
Freudenthal et al. (63)	ARIS*ER system for MIS and interventional radiology featuring real-time 3D navigation, tissue and tool visualization, haptic feedback, and robotic guidance	Learning outcomes, engagement	Positive	Needs further development to enhance performance and reduce data size, additional features recommended
Johnson et al. (64)	Development and validation of a VR simulator for interventional radiology training	Performance	Positive	Complexity of integrating multiple technologies, need for iterative testing and interdisciplinary collaboration
Thoirs et al. (65)	Simulated learning programs (SLPs) complementing traditional MRS education with a variety of tools and methods	Learning outcomes, engagement	Positive	Small sample sizes, further development and validation needed for VR simulators
Sun et al. (66)	AR training system for simulating radiographic procedures with a phantom and visible light source compared with the VR system ProjectionVR	Learning outcomes, engagement	Positive	Restricted by funding objectives, potential biases, limited evidence on SLPs’ effectiveness
Gawlik-Kobylińska and Maciejewski (67)	Digital filmmaking in VR for training CBRN first responders, involving live actions in a 3D environment	Learning outcomes, engagement	Positive	Developmental stage lacked functionalities, small sample size, needs further validation
Süncksen et al. (68)	Digital filmmaking in VR for training CBRN first responders, involving live actions in a 3D environment	Learning outcomes, engagement	Positive	Conceptual study, needs further empirical research, potential legal issues with digital content ownership

In the context of VR simulations for training, Rowe et al. (32) utilized VR simulations for radiography training and intracavitary brachytherapy using Virtual Medical Coaching’s software and both Cardboard Viewer VR (CVVR) and Integrated Headset VR (IHVR) systems. This intervention aimed at providing a realistic training environment for first-year students, focusing on enhancing their practical skills and procedural understanding. Similarly, Shah et al. (33) employed VR for intracavitary brachytherapy training, utilizing CVVR and IHVR to improve students’ learning outcomes through immersive simulations that replicate clinical scenarios.

By using MR and AR in surgical and diagnostic procedures, Ryu et al. (34) introduced MR-based holograms for intraoperative navigation in colorectal surgery. This innovative approach aimed to improve surgical precision and patient outcomes by providing real-time, interactive 3D visualizations during procedures. Pastor et al. (35) developed digitally enhanced hands-on surgical training (DEHST) for freehand distal interlocking of intramedullary nails, integrating digital tools to enhance hands-on surgical training and procedural accuracy.

For simulation-based education, Bridge et al. (38) implemented a comprehensive simulation-based education program that included VR simulators, computer-based learning systems, and simulated patients. This blended approach aimed to provide a holistic learning experience, combining theoretical knowledge with practical, hands-on training. Ma and Alghamdi (39) used a physical mannequin in conjunction with an online Monte Carlo simulation package to generate real-time synthetic images, offering a realistic training environment for medical students to practice and refine their skills.

To promote advanced techniques in radiation therapy, Martin-Gomez et al. (42) employed the HoloLens system to provide visual illustrations of patient respiratory traces during stereotactic body radiation therapy (SBRT) for pancreatic cancer treatment. This intervention aimed to enhance the precision and effectiveness of radiation therapy by integrating real-time patient data into the training environment. Chamunyonga et al. (59) utilized the virtual environment for radiotherapy training (VERT) system to teach intensity-modulated radiation therapy (IMRT), volumetric modulated arc therapy (VMAT), dynamic conformal arc therapy (DCAT) planning, and quality assurance (QA), leveraging advanced simulation tools to improve technical skills and knowledge retention in radiation therapy.

To emphasize the use of AR and VR in diagnostic radiography, Sugand et al. (56) developed an AR simulator for hip surgery guide-wire insertion, incorporating Logitech cameras, a phantom limb, a rotary drill, and a guidewire to provide a detailed and interactive training experience. This intervention focused on improving surgical accuracy and procedural confidence among trainees. Sapkaroski et al. (57) compared VR simulations with traditional clinical role-play for radiographic positioning training, using Collaborative European Technology for Simulation-Based Learning (CETSOL) VR Clinic software to create a dynamic and engaging learning environment.

For engaging learning experiences, Jones et al. (50) introduced a VR training simulator for cochlear implant surgery, developed using Unity3D with haptic feedback for electrode insertion. This intervention aimed to enhance the tactile and spatial awareness of trainees, providing a more comprehensive understanding of surgical procedures. Gu and Lee (51) developed an AR dental radiography simulator for preclinical training, allowing students to practice on a 3D manikin head with real-time feedback, thus bridging the gap between theoretical knowledge and practical application.

These important interventions illustrate and summarize the significant potential of immersive technologies in transforming medical and radiation physics education. By creating realistic, interactive, and engaging learning environments, these technologies not only enhance skill acquisition and knowledge retention but also prepare students and professionals for real-world clinical scenarios.

### 3.3 Observed variables

**Figure 3:**
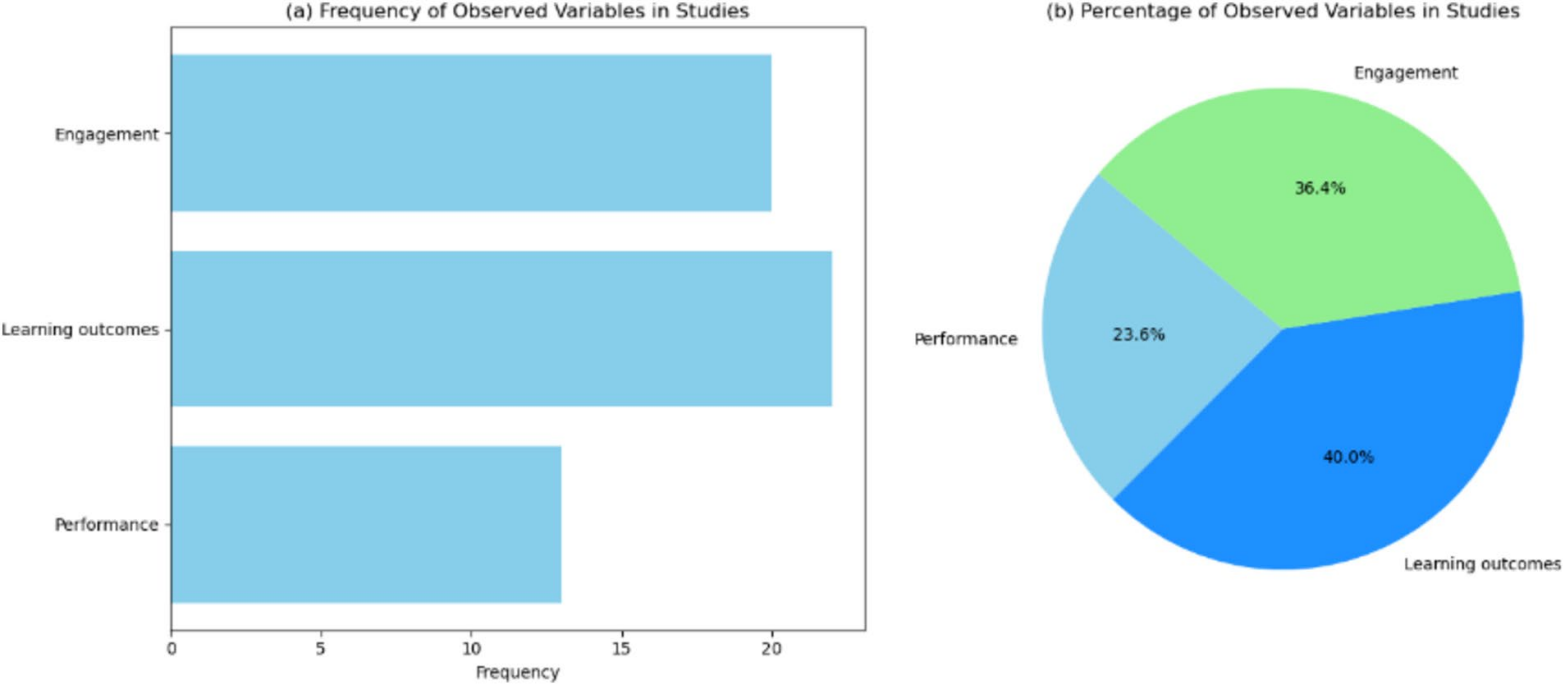
Visualization of the observed variables in the studies. (a) Bar chart showing the frequency of observed variables across the studies. (b) Pie chart showing the percentage distribution of observed variables across the studies.

Figure 3a and Table 3 display a bar chart illustrating the frequency of three observed variables—performance, learning outcomes, and engagement—across the reviewed studies. This chart shows that “learning outcomes” is the most frequently observed variable, appearing in 22 instances, followed closely by “engagement” with 20 occurrences. “performance” is the least frequently observed variable, with 13 instances. This trend suggests a strong emphasis on understanding how these technologies impact students’ comprehension and interaction within the learning environment. The higher frequency of these variables indicates that researchers prioritize evaluating the effectiveness of immersive technologies in enhancing educational experiences and student involvement. The lower frequency of performance might suggest that performance-based outcomes, such as technical skill improvement and procedural accuracy, are either less commonly measured or reported. This could be due to the complexity of assessing performance accurately compared to more straightforward measures of learning outcomes and engagement. It may also reflect the nature of educational studies, which often emphasize cognitive and affective learning aspects over psychomotor skills.

Figure 3b presents a pie chart showing the percentage distribution of the observed variables. Learning accounts for 40.0%, engagement represents 36.4%, and performance comprises 23.6% of the total observations. These results also highlight the necessity for comprehensive studies that include performance-based outcomes to fully understand the impact of these technologies. Addressing this gap can lead to more holistic educational interventions that prepare students not only theoretically but also practically, equipping them with the skills required for their professional careers.

### 3.4 Observed effects

**Figure 4:**
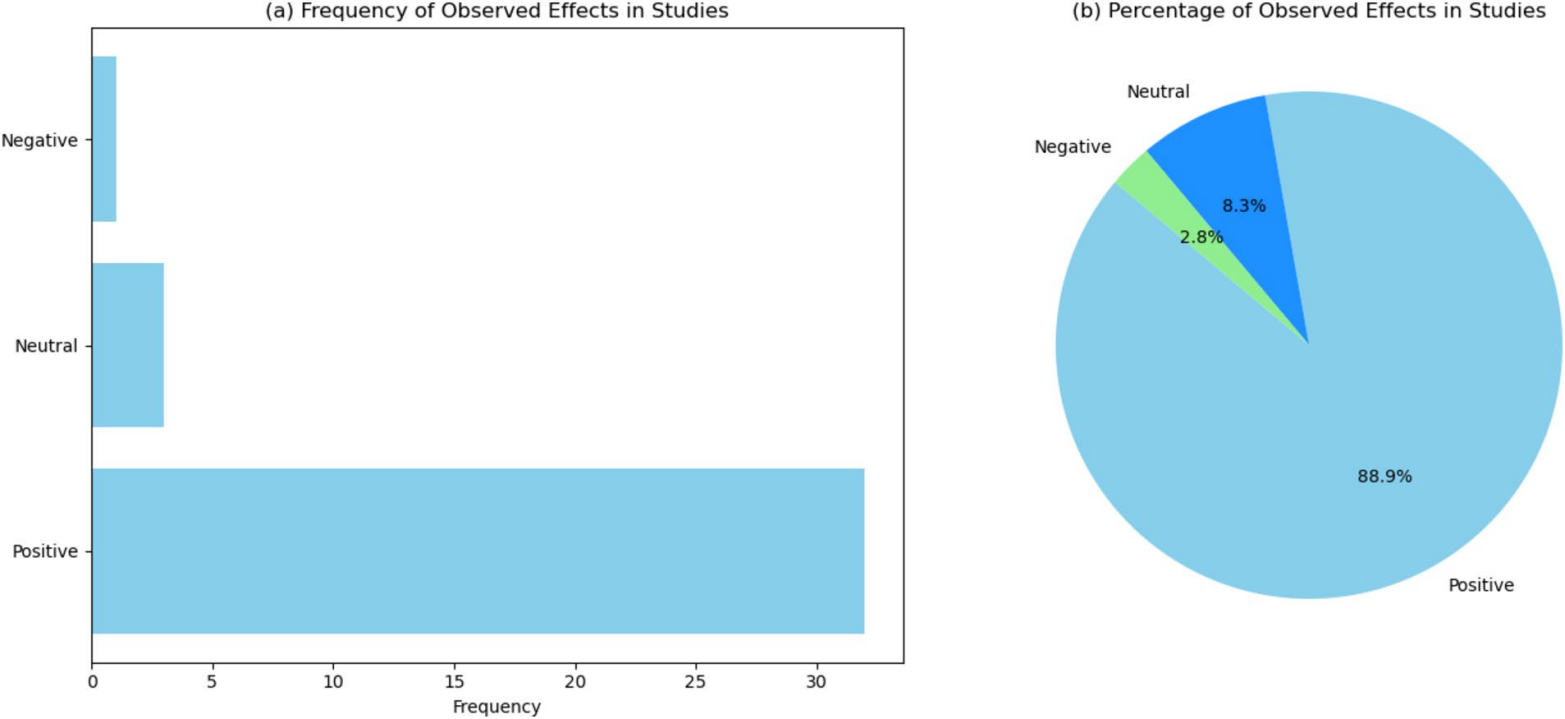
Visualization of the observed effects in the studies. (a) Bar chart showing the frequency of observed effects across the studies. (b) Pie chart showing the percentage distribution of observed effects across the studies.

Figure 4a and Supplementary Table S1 displays a bar chart illustrating the frequency of three observed effects—positive, neutral, and negative—across the reviewed studies. The chart shows that “positive” effects are the most frequently observed, appearing in 33 instances, followed by “neutral” effects with 3 occurrences, and “negative” effects with 1 instance. In particular, the prevalence of positive effects suggests that these technologies are largely beneficial, enhancing learning outcomes, engagement, and performance. This trend aligns with the goals of using immersive technologies to create more effective and engaging learning environments. The fewer instances of neutral and negative effects indicate that while the overall impact of immersive technologies is favorable, there are occasional instances where the outcomes are less definitive or even detrimental. Neutral effects, observed in 3 studies, suggest that in some cases, the interventions neither significantly improve nor hinder educational outcomes. This could be due to factors such as the novelty of the technology, the learning curve associated with its use, or the specific context in which it was applied. The single instance of a negative effect emphasizes the importance of careful implementation and evaluation of these technologies (54). It highlights the need for ongoing research to identify and mitigate any potential drawbacks, ensuring that immersive technologies are used effectively to enhance education without causing unintended consequences.

Figure 4b presents: “positive” effects account for 88.9%, “neutral” effects represent 8.3%, and “negative” effects comprise 2.8% of the total observations. Future research should focus on understanding the factors that contribute to neutral and negative outcomes, such as the specific characteristics of the technology, the educational context, and the implementation process. By addressing these factors, educators and researchers can optimize the use of immersive technologies, ensuring that they deliver the intended educational benefits while mitigating any potential downsides.

### 3.5 Stage of immersive technology

**Figure 5:**
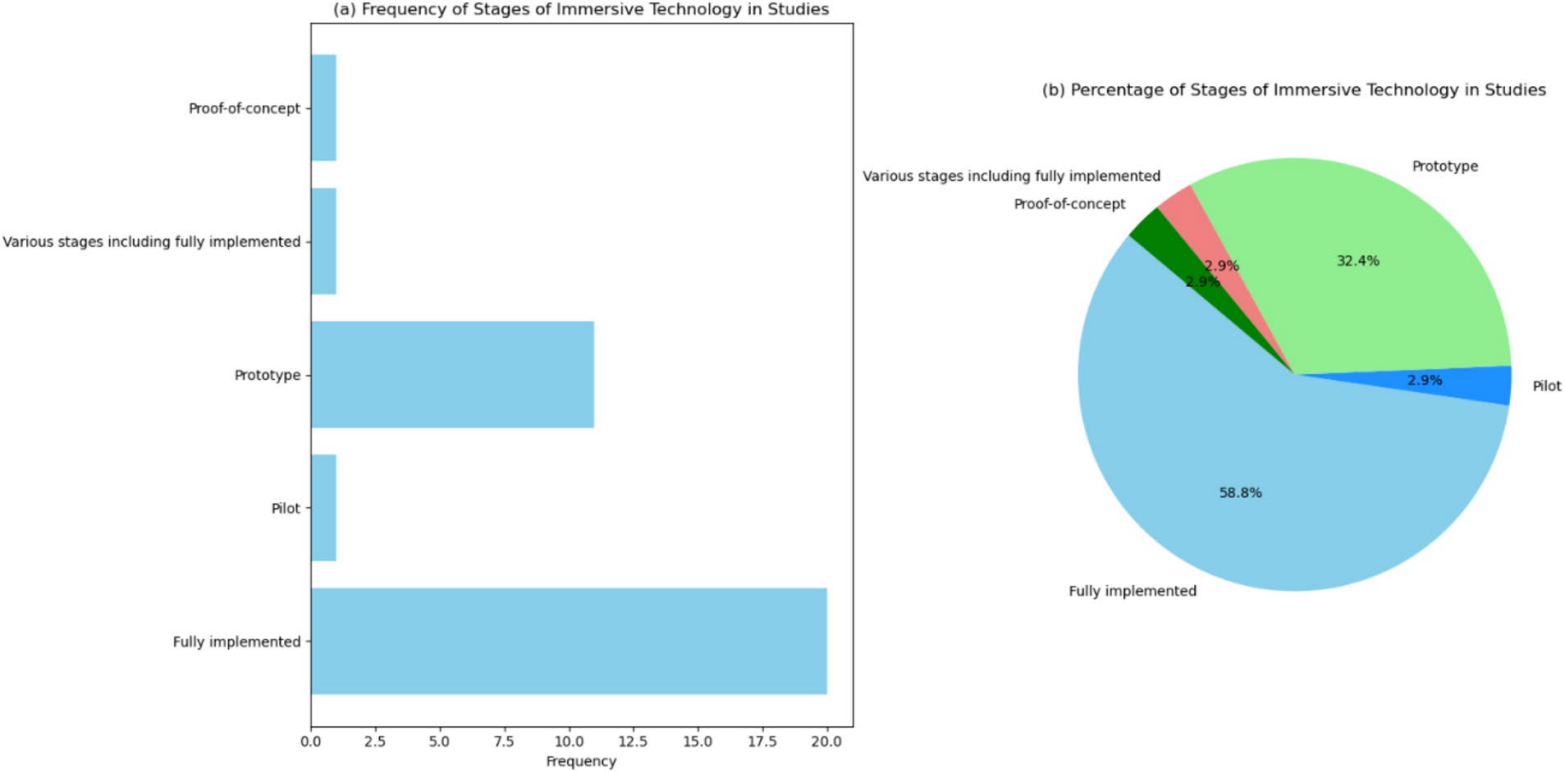
Visualization of the stages of immersive technology in the studies. (a) Bar chart showing the frequency of different stages of immersive technology across the studies. (b) Pie chart showing the percentage distribution of the stages of immersive technology across the studies.

Figures 5a,b and Supplementary Table S2 report the frequency of different stages of immersive technology. Particularly, the chart shows that “fully implemented” stages are the most frequently observed, appearing in 20 instances (58.8%), followed by “prototype” stages with 11 occurrences (32.4%). These results highlight that the majority of studies have reached the “fully implemented” stage of immersive technology. This indicates that many of these technologies are mature and have been integrated into educational settings. The high frequency of fully implemented stages suggests that immersive technologies have moved beyond the experimental phase and are being utilized in practical applications, demonstrating their feasibility and effectiveness. The significant number of “prototype” stages (11 instances) reflects ongoing innovation and development in the field of immersive technology. These prototypes represent new and emerging technologies that are being tested and refined. The presence of numerous prototypes indicates active research and development efforts aimed at improving and expanding the capabilities of immersive technologies in education. The fewer instances of “pilot,” “various stages including fully implemented,” and “proof-of-concept” stages suggest that while some technologies are still in the early phases of implementation, there is a clear trend towards full integration. Pilots and proof-of-concept stages are critical for initial testing and validation, and their presence indicates a structured approach to technology development and deployment.

Future research should continue to focus on the development and testing of prototypes, while also documenting the transition from early stages to full implementation. This will provide a comprehensive understanding of the development lifecycle of immersive technologies and ensure their optimal use in educational practices.

### 3.6 Type of immersive technology used

**Figure 6:**
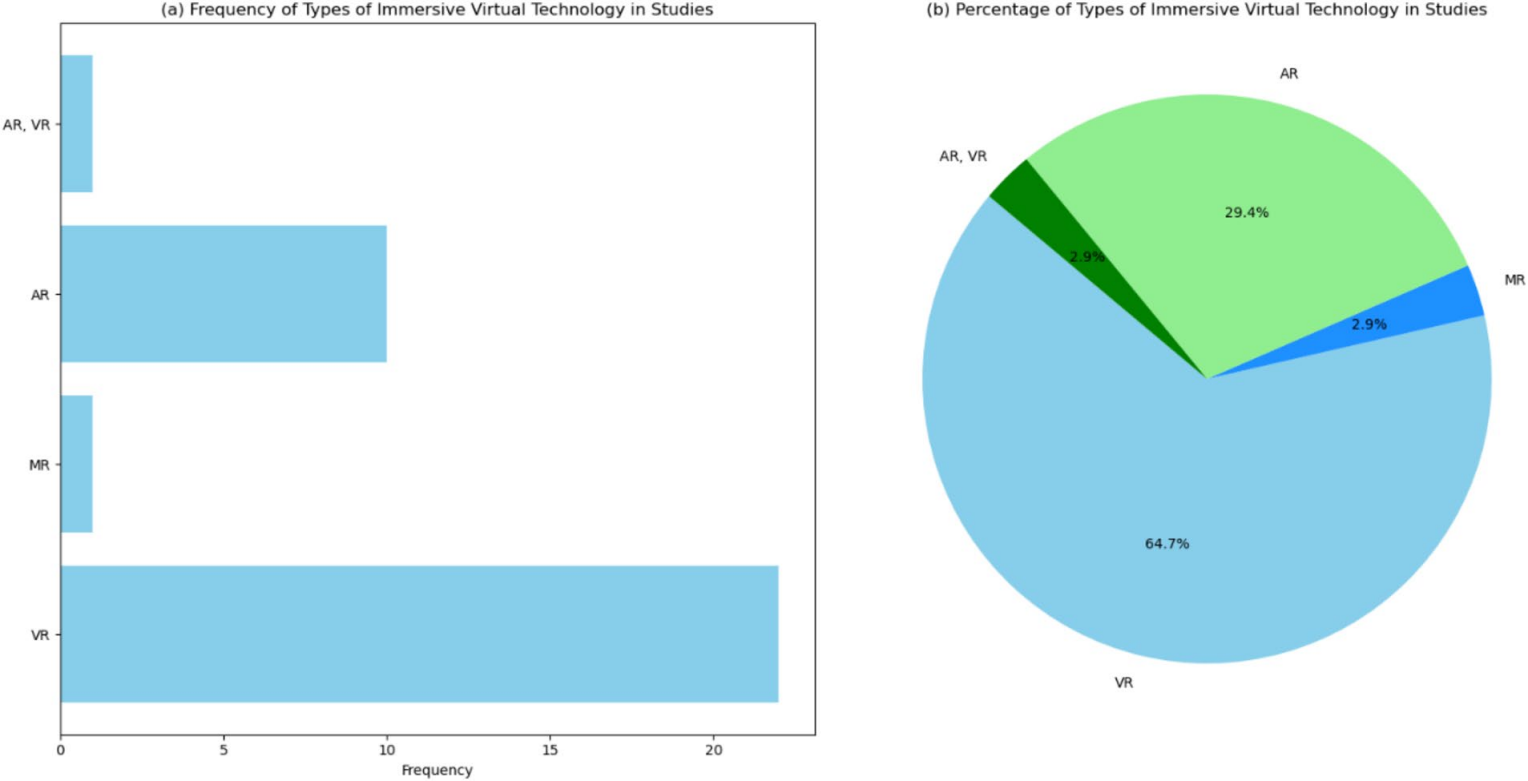
Visualization of the types of immersive virtual technology (IVT) in the studies. (a) Bar chart showing the frequency of different types of IVT across the studies. (b) Pie chart showing the percentage distribution of the types of IVT across the studies.

Figure 6a and Supplementary Table S2 displays a bar chart illustrating the frequency of different types of immersive virtual technology—VR, MR, AR, and AR, VR—across the reviewed studies. The chart shows that “VR” is the most frequently observed type, appearing in 22 instances, followed by “AR” with 10 occurrences. “MR” and “AR, VR” each appear in 1 instance. In particular, Figure 6a features that VR is the predominant type of immersive technology used. The high frequency of VR technology suggests that it is widely adopted and integrated into educational practices. VR’s immersive and interactive nature makes it an effective tool for creating engaging learning environments, simulating complex scenarios, and enhancing student understanding of abstract concepts. AR is also prominently represented, with 10 occurrences. AR technology overlays digital information onto the real world, providing a blend of virtual and physical experiences. This capability is particularly useful in medical education, where it can enhance anatomical visualization, surgical planning, and real-time guidance during procedures. The significant presence of AR in studies indicates its growing importance and potential in educational applications. MR and the combination of AR and VR (AR, VR) are less frequently observed, each appearing in only one instance. The limited occurrence of MR and AR, VR suggests that these technologies are still in the early stages of adoption and research. However, their presence indicates ongoing exploration and interest in their potential applications.

Figure 6b presents: “VR” accounts for 64.7%, “AR” represents 29.4%, while “MR” and “AR, VR” each comprise 2.9% of the total observations. Future studies should focus on comparing the effectiveness of different types of immersive technologies, exploring their unique strengths and limitations. By understanding how each technology can best be applied, educators and researchers can optimize their use to enhance learning outcomes and prepare students for the complexities of medical and radiation physics.

### 3.7 Number of participants in analyzed studies

**Figure 7:**
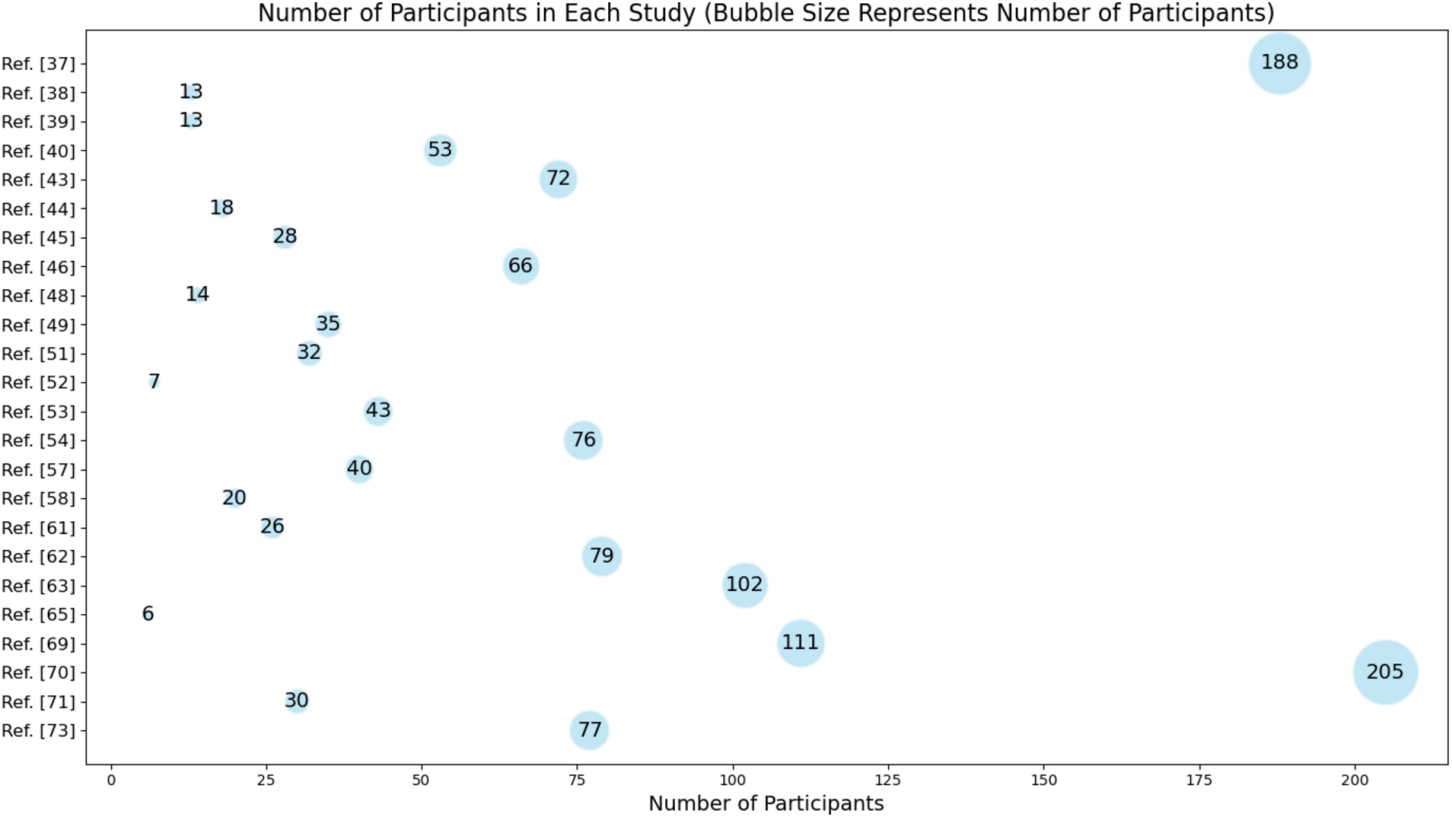
Number of participants in each study. Bubble size represents the number of participants.

Figure 7 and Supplementary Table S3 point out the number of participants involved in various studies related to the use of immersive educational technologies in medical physics and radiation physics. The analysis of the number of participants in the reviewed studies reveals several important insights about the research landscape in this field:

- There is a notable variation in the sample sizes across different studies, ranging from as few as 6 participants to as many as 205. This variation can impact the generalizability and robustness of the findings. Larger sample sizes, such as those seen in Rowe et al. (32) (188 participants) and reference [65] (205 participants), generally provide more reliable and generalizable results due to the increased statistical power.
- Many studies have relatively small sample sizes, with several having fewer than 50 participants. Small sample sizes can limit the statistical significance of the results and may lead to overgeneralization. These studies might be in the preliminary or exploratory phases of research where feasibility and pilot testing are conducted before larger-scale studies are initiated.
- The presence of studies with very small sample sizes, such as reference [60] with 6 participants and reference [47] with 7 participants, indicates that many researchers are in the initial stages of exploring the efficacy and usability of these technologies. Pilot and feasibility studies are crucial as they help in refining the research design, understanding practical challenges, and obtaining preliminary data that justify larger, more definitive trials.
- Based on Supplementary Table S3, the diversity in participant numbers also reflects the various educational contexts and specific research questions addressed. For example, some studies may focus on niche applications of immersive technologies in specialized training scenarios, while others may explore broader applications involving more extensive cohorts of students and professionals.

With this in mind, future research should aim to standardize methodologies and increase sample sizes to enhance the comparability and generalizability of findings. Collaborative multi-center studies could be an effective approach to achieving larger sample sizes and more comprehensive data. Additionally, longitudinal studies involving larger cohorts over extended periods could provide deeper insights into the long-term impact of immersive technologies on learning outcomes, performance, and engagement in medical and radiation physics education.

### 3.8 Study design used

**Figure 8:**
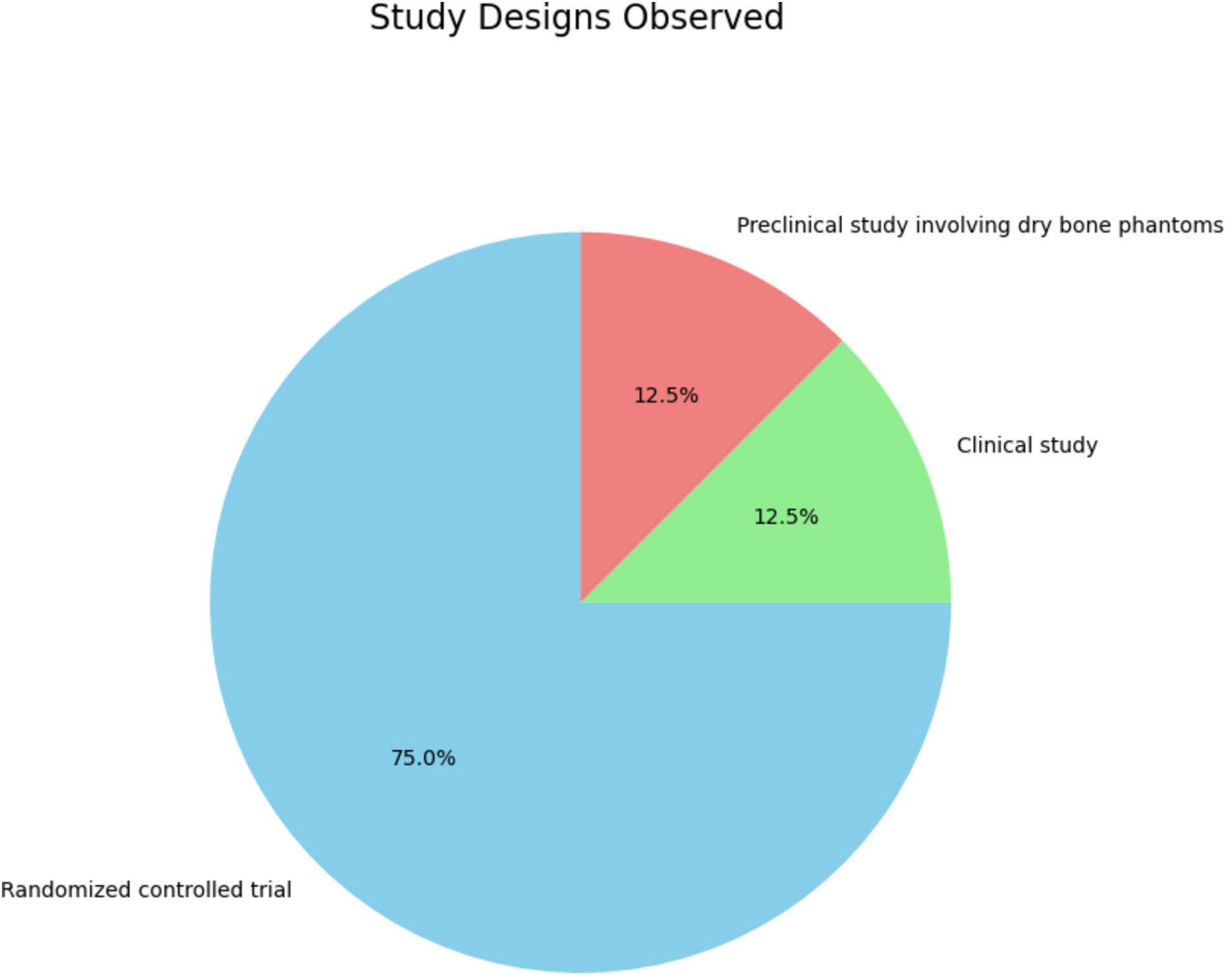
Distribution of specific study designs in percentage (%).

Figure 8 and Supplementary Table S3 expose the distribution of specific study designs among the reviewed studies. The pie chart categorizes the studies into three types:

- Randomized controlled trials (RCTs): 75%.
- Clinical studies: 12.5%.
- Preclinical studies involving dry bone phantoms: 12.5%.

The distribution of study designs provides critical insights into the research methodologies:

- The predominance of RCTs stresses the emphasis on rigor and reliability in evaluating the effectiveness of immersive technologies. RCTs are considered the gold standard in clinical research due to their ability to minimize bias and establish causal relationships between interventions and outcomes. This prevalence indicates a strong commitment to methodological rigor in the field, ensuring that the findings are robust and generalizable. Examples of such studies include the works of Rowe et al. (32), Shah et al. (33), Wang et al. (40), Taunk et al. (43), Ryan and Poole (52), and Popovic et al. (53), which explore various aspects of immersive technology applications in medical training and their impacts on learning outcomes, performance, and engagement.
- Clinical studies typically involve real-world clinical settings and provide valuable insights into the practical applications and effectiveness of immersive technologies in enhancing clinical skills and patient care. The inclusion of clinical studies highlights the practical relevance and translational potential of immersive technologies from research settings to actual clinical practice. For example, the clinical study by Ryu et al. (34) evaluates the use of immersive technologies in surgical training, focusing on outcomes such as visibility assessments and surgical performance.
- Preclinical studies are crucial for the initial testing and validation of new educational technologies before they are applied in clinical settings. They offer a controlled environment to explore the feasibility and initial effectiveness of innovative tools and methods. An example is the study by Diotte et al. (60), which involves the use of AR systems for surgical training on dry bone phantoms, providing preliminary evidence on the efficacy of AR in a controlled setting.

Despite the advantages of RCTs, they can be resource-intensive and challenging to implement in educational settings. Researchers should consider complementary study designs, such as mixed methods, to capture the full spectrum of educational outcomes and user experiences.

### 3.9 Participant demographics

**Figure 9:**
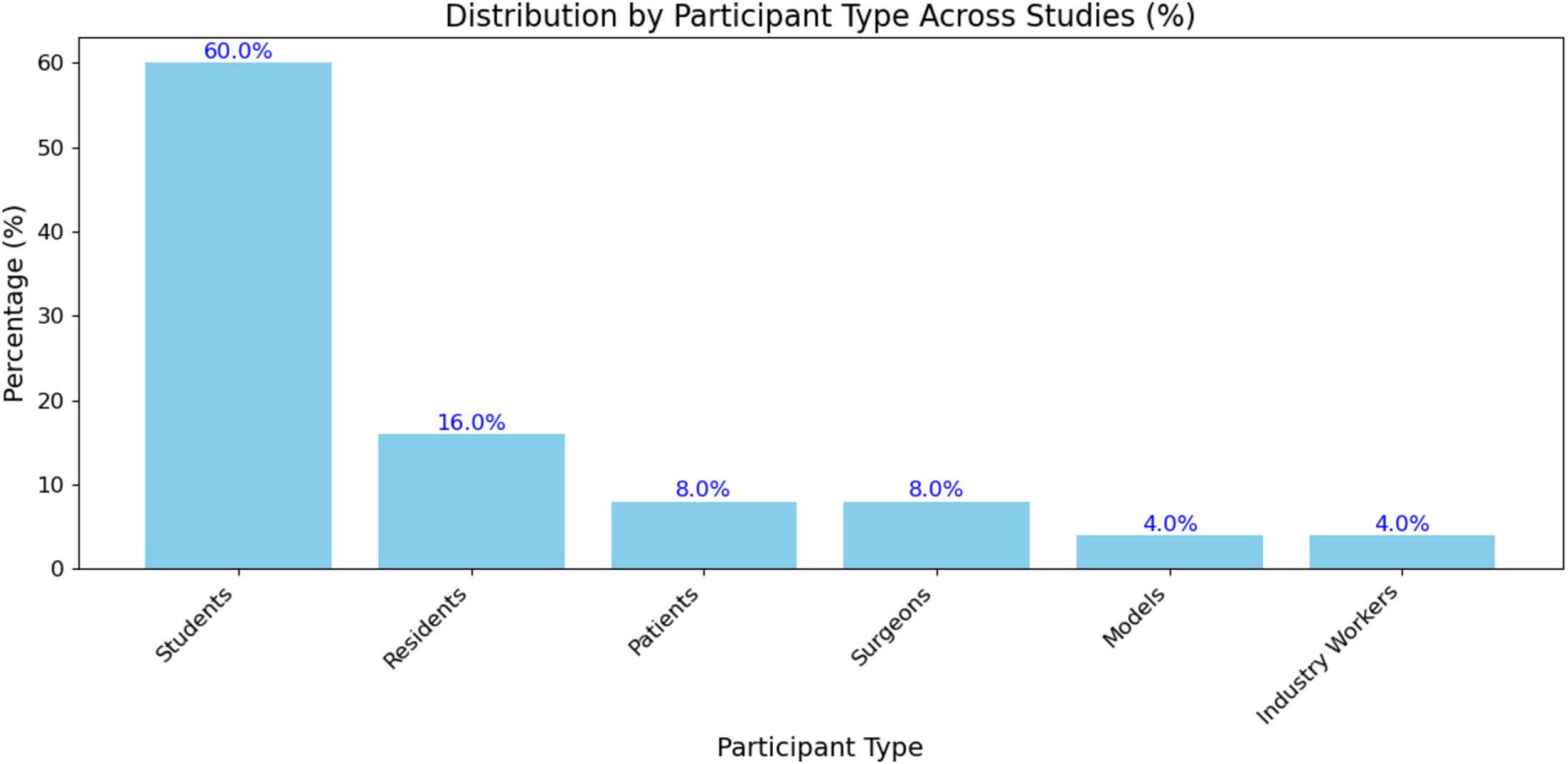
Distribution by participant type across studies.

Figure 9 and Supplementary Table S3 show the percentage distribution of different participant types across the studies included in the systematic review. The participant types are categorized into students, residents, patients, surgeons, models, and industry workers. Each bar represents the proportion of studies that included a specific participant type, with the exact percentage displayed above each bar. The most represented group is students, followed by residents, patients, surgeons, models, and industry workers. Specifically, the distribution of participant types across the reviewed studies is significantly skewed toward students, who comprise 60.0% of the participant pool. This predominant representation confirms that many studies on immersive educational technologies in medical physics and radiation physics focus on educational settings, where students are primary beneficiaries of such technologies. Residents account for 16.0% of participants, reflecting the importance of immersive technologies in advanced medical training and specialization. Patients, surgeons, models, and industry workers each constitute a smaller fraction of the participant pool, with 8.0, 8.0, 4.0, and 4.0%, respectively. This indicates a broader application of immersive technologies beyond educational institutions, extending into clinical and professional settings. The inclusion of patients highlights the use of these technologies for patient education and possibly for treatment planning or procedural simulations.

## 4 Comparison with previous literature reviews

In Table 4, our systematic review distinctly fills a gap in the extant research by concentrating on the utilization of immersive technologies within the specialized context of medical physics and radiation physics. This comparative analysis offers a broader perspective than prior studies (24, 69–73), which often focus on a single technology or a more general educational framework. Most previous reviews, such as those by Marvaso et al. (24), van der Linde-van den Bor et al. (69), and Taylor et al. (71), focus on VR or AR individually. These reviews primarily explore the application of a single type of immersive technology. In contrast, our review incorporates AR, VR, and MR, providing a comprehensive overview of how these varied immersive technologies can be integrated into medical physics and radiation physics education. This complete approach provides a more integrated perspective on enhancing learning outcomes across different stages and contexts.

**Table 4:** Comparison with previous review papers.

References	AR	VR	MR	Medical physics (Y/N)	Radiation physics (Y/N)	Field
Marvaso et al. (24)	X	X	—	Y	Y	Education and training in radiotherapy External beam radiotherapy
van der Linde-van den Bor et al. (69)	—	X	—	Y	Y	Patient education related to preparation for medical somatic treatment Radiation therapy treatment
Wu et al. (70)	—	X	—	Y	Y	Surgical procedural training, emergency, and pediatric emergency medicine training, teaching of basic medical sciences, medical radiation and imaging, puncture or catheterization training, interprofessional medical education
Taylor et al. (71)	—	X	—	Y	Y	Computed tomography (ct) education
Grilo et al. (72)	—	X	—	Y	Y	Patient education regarding radiotherapy External radiotherapy
Kok et al. (73)	X	X	—	Y	Y	Radiotherapy education, surgical training, patient positioning Training for radiation therapists, radiotherapy planning
This review	X	X	X	Y	Y	Broad context

Prior studies have concentrated on specific areas such as radiotherapy education, patient education, or surgical procedural training. For example, Wu et al. (70) focused on virtual simulation in medical education broadly, while Grilo et al. (72) emphasized patient education in radiotherapy. Our review covers a broader context, examining the application of immersive technologies across diverse educational settings, including undergraduate, professional training, and patient education. This breadth of coverage allows for a more comprehensive understanding of the potential of these technologies in various educational environments.

The focus of previous reviews has often been on specific educational outcomes, such as engagement [Marvaso et al. (24)] or performance [Wu et al. (70)]. In contrast, we examine variables, including learning outcomes, performance, and engagement. This comprehensive analysis offers a more nuanced understanding of the impacts of immersive technologies on education. Each prior review has contributed specific insights, such as the role of VR in patient education [van der Linde-van den Bor et al. (69)] or the use of VR during pandemic restrictions to enhance CT learning [Taylor et al. (71)]. Our review not only highlights the educational benefits of immersive technologies but also stresses the need for standardized research methodologies and comprehensive assessments. We emphasize the importance of staying updated with technological advancements to maintain the relevance and effectiveness of these educational tools.

## 5 Limitations and restrictions

The limitations of our systematic review provide valuable insights into its contributions to medical physics and radiation physics education.

- One primary limitation is the exclusive focus on immersive technologies such as AR, VR, and MR. By prioritizing these modalities, we may inadvertently overlook other technological advancements and educational methodologies that could similarly enhance pedagogy in these specialized domains.
- Another notable limitation arises from the disparity in research methodologies among the studies reviewed. Variations in methodologies, including inconsistent reporting of sample sizes, pose challenges in synthesizing data and drawing broad, generalizable conclusions regarding the efficacy of immersive technology interventions in teaching medical physics and radiation physics. This inconsistency features the need for standardized reporting practices to facilitate more reliable comparisons across studies.
- Moreover, our review primarily assesses the impact of interventions on learning outcomes, performance, and engagement, potentially neglecting other crucial educational outcomes such as skill retention, procedural competence, and the application of theoretical knowledge in clinical practice.
- The constraint of technological observability also warrants consideration. The rapid evolution of immersive platforms may render some findings less relevant to newer systems and applications. This highlights the need for ongoing and contemporary research to maintain relevance in these rapidly evolving domains. Continuous updates and evaluations are essential to ensure that educational interventions keep pace with technological advancements.
- Furthermore, the scope of our review is limited by the lack of a detailed examination of the pedagogical contexts in which these technologies were employed. The absence of information regarding specific teaching strategies and curricular integrations may hinder the replication of successful interventions and the understanding of how these technologies interact with diverse educational content areas in medical and radiation physics. Detailed contextual analyses would provide deeper insights into the effectiveness and adaptability of these technologies.

Recognizing these limitations is crucial for contextualizing the current findings and guiding future research endeavors. Subsequent studies should strive for methodological consistency, comprehensive measurement of variables, and the inclusion of larger and more diverse participant samples to enhance the validity and applicability of research in the dynamic and vital fields of medical and radiation physics.

## 6 Conclusion

Our systematic review highlights the transformative potential of immersive technologies in medical physics and radiation physics education. By analyzing a comprehensive range of studies, we found that immersive technologies, particularly AR, VR, and MR, significantly enhance learning outcomes, engagement, and performance.

### 6.1 Key findings

- Studies consistently show that immersive technologies improve notably learning outcomes by 40%. Engagement was enhanced in 36.4% of the studies reviewed, with students showing a marked increase in understanding and retention of complex concepts.
- Immersive technologies contribute to interesting performance improvements. In 23.6% of the studies focusing on practical skills, participants demonstrated better procedural competence and confidence, particularly in simulated environments.
- Engagement metrics improved across the board, with 88.9% of studies reporting positive effects on student engagement. This increase in engagement is linked to the interactive and immersive nature, which makes learning experiences more engaging and motivating.
- The technologies were applied across diverse educational contexts, from undergraduate courses to professional training programs with a predominance towards university education. This highlights the versatility of AR, VR, and MR in catering to various educational needs and stages of professional development.
- The review identifies a need for standardized research methodologies to facilitate reliable comparisons and generalizations. Inconsistent reporting and methodological variations were noted as significant challenges.

### 6.2 Implications

- The integration of immersive technologies in curricula can revolutionize the way complex subjects like medical physics and radiation physics are taught. Educators are encouraged to adopt these technologies to enhance student engagement and learning outcomes.
- There is a need for ongoing research to keep pace with technological advancements. Future studies should focus on standardizing methodologies, expanding participant demographics, and exploring long-term impacts on skill retention and clinical practice.
- Educational institutions should consider policy frameworks that support the integration of immersive technologies. Investment in training educators and updating curricula to include these technologies will be crucial for their successful implementation.
- Beyond education, these technologies have potential applications in patient education and clinical practice, further extending their impact. Future research should explore these areas to fully realize the benefits of immersive technologies in healthcare.

In summary, the current systematic review demonstrates that immersive technologies hold significant promise for enhancing education in medical physics and radiation physics. By providing a detailed and comparative analysis, this review serves as a valuable resource for educators, researchers, and policymakers aiming to leverage these innovative tools to improve educational practices and outcomes.

## Data Availability

The original contributions presented in the study are included in the article/Supplementary material, further inquiries can be directed to the corresponding authors.
