# Supplementary material for: A systematic review of immersive educational technologies in medical physics and radiation physics
Source: Front Med (Lausanne). 2024 Oct 10;11:1384799. doi: 10.3389/fmed.2024.1384799 (PMC11499124; doi:10.3389/fmed.2024.1384799)
Supplement: Supplementary file 1 [file Data_Sheet_1.docx]

**Supplementary Information: A Systematic Review of Immersive Educational Technologies in Medical Physics and Radiation Physics**

**Talia Tene^1,^*, Nataly Bonilla García^2^, Diana Coello-Fiallos^2^, Myrian Borja^3^, Cristian Vacacela Gomez^4,^***

^1^Department of Chemistry, Universidad Técnica Particular de Loja, 110160 Loja, Ecuador

^2^Facultad de Ciencias, Escuela Superior Politécnica de Chimborazo (ESPOCH), Riobamba 060155, Ecuador

^3^Grupo de Investigación Ciencia de Datos (CIDED), Escuela Superior Politécnica de Chimborazo (ESPOCH), Riobamba 060155, Ecuador

^4^INFN-Laboratori Nazionali di Frascati, Via E. Fermi 54, I-00044 Frascati, Italy

*** Correspondence:**T. Tene

[tbtene@utpl.edu.ec](mailto:tbtene@utpl.edu.ec)

C. Vacacela Gomez
[vacacela@lnf.infn.it](mailto:vacacela@lnf.infn.it)

**Table S1.** Interventions considering the variable and related effects

| **Reference** | **Intervention** | **Variable** | **Effect** | **Barriers/Challenges** |
| --- | --- | --- | --- | --- |
| Rowe, D., et al. 2023 [37] | VR simulation for radiography training using Virtual Medical Coaching’s software, and VR simulation for intracavitary brachytherapy using CVVR and IHVR. | OSCE duration, machinery movement errors, radiographic exposure errors, and patient positioning errors | Significant reduction in OSCE duration and equipment/patient positioning errors in the VR group. No difference in radiographic exposure errors. | The study does not mention specific barriers or challenges faced during the intervention |
| Shah et al., 2022 [38] | VR simulation for intracavitary brachytherapy training using cardboard viewer VR (CVVR) and integrated headset VR (IHVR) | Self-confidence, procedural knowledge, perceived usefulness, objective proficiency metrics | Improvement in self-confidence, similar proficiency metrics, cost-effectiveness of CVVR | Technical difficulties with CVVR, battery life issues with IHVR |
| Ryu et al., 2022 [39] | Mixed reality-based hologram for intraoperative navigation in colorectal surgery | Hologram visibility, surgical outcome, NASA Task Load Index (TLX) scores | Improved understanding of anatomy, safe surgery with holograms, various TLX scores | Not specified |
| Pastor et al., 2022 [40] | Digitally Enhanced Hands-On Surgical Training (DEHST) for freehand distal interlocking of intramedullary nails | Number of X-rays, nail hole roundness, drill tip position, drill hole accuracy, face validity questionnaire | Higher number of X-rays for novices, higher hit/miss ratio for experts, high realism and training capability ratings | Some participants noted the drill power tool was too light, bone too soft, C-arm size too small |
| Chen et al., 2022 [41] | VRContour, a VR-based tool for contouring medical structures. | Skill acquisition, usability, task load, attention allocation. | High similarity in contours between participants and experts (DSC scores), efficient task completion, improved focus on 3D structures. | Difficulty with depth perception and fine-grained control in 3D drawing. |
| Kiryukhin et al. 2022 [42] | Virtual analog of uranium-water subcritical assembly for education and training | Learning effectiveness, engagement, understanding of subcritical assembly concepts | Enhanced understanding, high engagement, improved learning outcomes | Not reported |
| Bridge et al., 2021 [43] | Simulation-based education (SBE) including VR simulators, computer-based systems, and simulated patients | Skill acquisition, confidence, patient safety awareness, problem-solving, teamwork, decision-making skills | Improved technical skills, increased confidence, better problem-solving and teamwork, enhanced patient safety awareness | Funding, access to resources, social distancing restrictions |
| Ma et al. 2011 [44] | Consists of a physical mannequin representing the patient, a physical model of the imaging modality, and an online Monte Carlo simulation package that generates synthetic images in real time | Skill acquisition Knowledge retention User engagement | Improved learning experience Better knowledge acquisition and retention compared to conventional lectures | System setup requiring time and great care. Need for training to maximize the benefit of the system tools |
| Wang et al., 2021 [45] | Unguided trauma simulation practice using the TraumaVision VR Simulator (Swemac) for distal locking screw placement | Task completion time, radiation use, success rate, overall quality score | Improved speed and success rates in the trained group, no significant improvement in radiation use or overall score | Not specified |
| Gunn et al., 2021 [46] | VR CT simulation for medical imaging (MI) and radiation therapy (RT) undergraduate students to learn CT scanning | Confidence in performing diagnostic and planning CT scans, engagement (usefulness, ease of use, enjoyment) | Increased confidence in performing CT scans, higher engagement leading to increased confidence | Technical difficulties, need for facilitator expertise, software familiarity |
| Martin-Gomez et al., 2020 [47] | AR system using HoloLens2 to provide visual feedback of patient's respiratory trace during SBRT for pancreatic cancer treatment | Achievement of air volume threshold, airflow rate, engagement, feedback effectiveness | Improved patient engagement, consistent achievement of air volume threshold, reduced intra-fraction variation, positive user feedback | Ergonomics, technology adoption, hardware autonomy, radiation tolerance, disinfection and sterilisation |
| Taunk et al., 2021 [48] | VR-based intracavitary brachytherapy simulation for gynecologic brachytherapy training | Self-confidence, procedural knowledge, perceived usefulness, technical skills, implant quality, time of implant | Increased confidence, improved technical skills, reduced time of implant, higher perceived usefulness | None specified |
| Czaplinski et al, 2020 [49] | Blended learning framework with VERT (Virtual Environment Radiotherapy Training) simulations for medical physics students | Student engagement, satisfaction, understanding of theory, practical skills application | Increased student engagement, improved satisfaction, enhanced understanding of theory, better practical skills applicatio | Technical difficulties, cognitive overload in VERT sessions |
| Kazuki Nishi et al., 2020 [50] | AR application for visualising the spread of scattered radiation in radiography using AR | Radiation dose estimation, understanding of radiation spread, user engagement | Improved understanding of radiation spread, effective radiation dose estimation, high engagement | Need for digital skills, technology adoption, hardware limitations |
| Kang et al., 2020 [51] | Gross anatomy laboratory sessions with AR tools for medical physics students | Student engagement, satisfaction, understanding of anatomy, comfort level with lab environment | Increased student engagement, improved satisfaction, enhanced understanding of anatomy, greater comfort with lab environment | Technical difficulties, cognitive overload in AR sessions, initial discomfort with cadaver labs |
| Johnson et al., 2020 [52] | 360-degree VR video outlining the technical aspects of EBRT to the pelvis as a supplement to traditional education methods | Understanding of treatment process, anxiety levels, information needs met | Increased understanding of treatment process, potential anxiety reduction, identified gaps in traditional education methods | Language, age, visual issues, realism and generalizability of video, overwhelming amount of visual information |
| Park et al., 2020 [53] | 3D AR visualization of preprocedural MR images for guiding transarterial embolization in a preclinical model of hepatocellular carcinoma | Fluoroscopy time, catheterization time, radiation dose (DAP and AK) | 37% reduction in fluoroscopy time, 27% reduction in catheterization time, no significant reduction in radiation dose | Technical setup time for AR, need for additional preprocedural planning |
| Sapkaroski et al., 2019 [54] | VR simulation using CETSOL VR Clinic software for radiographic positioning training versus traditional clinical role-play | Student perception of skill acquisition, engagement, comparability to clinical practice | No significant difference in perception scores between VR and role-play; VR allowed more repetition and self-paced learning | Calibration issues with VR setup, limited patient interaction in VR |
| Jones et al., 2019 [55] | VR training simulator for cochlear implant surgery developed using Unity3D with haptic feedback for electrode insertion | Insertion time, number of successful insertions, failed attempts, average insertion depth, total time spent training, number of simulator resets | No significant differences between simulated and actual electrode insertion in terms of deformation angles, consistent performance metrics | Technical setup time for VR and haptic device, need for high computational performance |
| Ja-Young et al., 2019 [56] | AR dental radiography simulator for preclinical training, allowing students to practice on a 3D manikin head using a mobile device with real-time feedback on positioning and technique. | Understanding of dental anatomy, skill acquisition in radiographic techniques, user engagement. | Improved understanding of dental anatomy, enhanced radiographic technique skills, high engagement levels. | Technical setup, need for iOS and Android compatibility, potential initial learning curve for using the AR application. |
| Ryan, E. et al., 2019 [57] | Virtual learning environment (VLE) for radiation therapy education | Student satisfaction, engagement, recall, and retention | Significant improvement in satisfaction and engagement, higher recall scores, no significant difference in retention | Potential bias due to the small sample size, possibility of the Hawthorne effect |
| Popovic, B, et al., 2019 [58] | Simulation training in coronary angiography using the Simbionix Angio-Mentor, focusing on catheter handling, fluoroscopic time, procedural time, and contrast amount, with both virtual and real-life practice. | Key variables measured include procedural time, radiation dose, global procedure skill score, fluoroscopic time, training contrast amount, and global training score. | Significant improvements in procedural time, radiation dose, skill score, fluoroscopic time, contrast amount, and training score, with enhanced skill transfer to real-life practice. | High costs of simulators, limited access to simulation centers, lack of standardized curriculum incorporating simulation |
| Fernández, C., et al., 2018 [59] | Use of virtual reality (VR) devices in front of the eyes to simulate real-world conditions and measure radiation absorption in the brain and eyes of children compared to adults | Specific Absorption Rate (SAR), tissue-specific radiation doses, and developmental trends in tissue absorption | Children absorb higher doses of radiation in the brain and eyes compared to adults when using VR devices. This highlights the need for refined regulatory compliance testing and public education on safe use. | Potential limitations of using anatomical models and simulations to represent real-world conditions |
| Guo, J., et al., 2018 [60] | Augmented Reality (AR) system for antenna design education, which enhances user interaction and engagement through visual and interactive methods using mobile phones, smart gloves, and virtual buttons. | User engagement, ease of mastering antenna design, interaction level | Enhanced user interest and engagement, improved visibility and understanding of antenna design, easier for beginners to master compared to traditional software | Current system not professional enough for advanced users, needs more functions to be added in the future |
| Sugand, K. et al., 2018 [61] | FluoroSim, an AR simulator for hip surgery guide-wire insertion using Logitech cameras, a phantom limb, a rotary drill, and a guide-wire for radiation-free simulation. | Tip-apex distance (TAD), cut-out rate (COR), procedural time, radiographs taken, guide-wire retries | Significant differences in TAD, COR, and radiographs taken among novice, intermediate, and expert surgeons, with experts and intermediates outperforming novices. Face and content validity demonstrated. | Surgeons' sense of urgency to complete the task due to daily responsibilities, potential influence on total procedural time |
| Sapkaroski, D., et al., 2018 [62] | CETSOL VR Clinic, a haptic feedback VR simulation for medical imaging students, using Oculus Rift and HTC Vive for dynamic patient interaction and clinical exams with hand gesture, gaze, and positional tracking. | Student perception scores, skill acquisition in patient positioning, x-ray tube centering, cassette alignment, image processing, and understanding of radiographic procedures. | Higher perception scores and significant improvement in clinical and technical skills with CETSOL VR Clinic compared to Shaderware. | Relied on student perceptions, did not measure clinical performance directly, and limited comparison to general procedural workflow without VR peripherals.. |
| Gunn, T., et al., 2017 [63] | VR simulation for training first-year medical imaging students in a virtual x-ray room with interactive equipment and patient, practicing radiographic positioning for the foot and scaphoid. | Technical skill acquisition, role-play skill scores, and demographic factors | Significant improvement in role-play skill scores for the VR group (mean score 30.67) compared to the traditional group (mean score 28.8). | Some data were excluded due to errors in data collection, and the study was limited by reduced cohort sizes. |
| Chamunyonga, C. et al., 2017 [64] | VERT system for teaching IMRT, VMAT, DCAT planning, and QA, evaluating dose coverage and OAR sparing with ArcCHECK and IMRT phantoms.Principio del formularioFinal del formulario | Student engagement, understanding of treatment planning concepts, skill acquisition in dose coverage evaluation, and QA procedures. | VERT improved students' ability to evaluate treatment plans and reinforced understanding of plan evaluation, QA, and treatment delivery skills. | Limited time and staff during teaching periods to develop a range of plans for export to VERT, the system's current inability to visualize tumor motion in real-time |
| Diotte, B., et al., 2014 [65] | AR fluoroscope for assisting surgeons in distal locking of intramedullary nails, integrating optical and X-ray images for accurate drill tip positioning with fewer X-ray acquisitions | Success rate, number of X-ray images, and average navigation time per hole. | Surgeons completed the distal locking procedure 98.3% of the time with a single X-ray image, with an average navigation time of 1.4 ± 0.9 minutes per hole. | Initial setup and calibration of the AR system, learning curve for using the augmented drill |
| Szőke, I., et al., 2014 [66] | VRdose and Halden Planner for 3D radiation risk assessment and work simulation in nuclear environments, supporting planning, briefing, training, and communication within teams and with external stakeholders. | Success rate, number of X-ray images, navigation time, and radiation dose assessment. | VRdose improved visualization of radiation fields, accurate dose calculation, risk estimation, and training efficiency, enhancing safety in nuclear environments. | Challenges included the need for extensive calibration and setup of the VR system and the integration of up-to-date dosimetric packages. |
| Nishi, K., et al. 2021 [67] | Development and use of a WebAR system to visualize scattered radiation during portable imaging, using the Monte Carlo method to simulate radiation behavior and displaying it in AR via web browsers for accessible radiation protection education. | Key variables measured include the effectiveness of the visualization in enhancing understanding of scattered radiation behavior, user engagement, and ease of use across different devices. | Digital filmmaking in VR for training CBRN first responders, involving live actions in a 3D environment and creating instructional videos using special software. | Issues with device compatibility and performance, such as differences in camera quality and operating systems, were noted. The system also faced challenges in ensuring smooth operation on lower-performance devices. |
| Freudenthal, A., et al, 2011. [68] | ARIS*ER system for MIS and interventional radiology, featuring real-time 3D navigation, tissue and tool visualization, haptic feedback, and robotic guidance. | Effectiveness of user interface, workflow integration, accuracy of 3D navigation, user satisfaction, and safety improvements. | Improved navigation and precision in surgical procedures, enhanced user satisfaction, better workflow integration, and reduced human error. | Technological limitations, extensive component integration, and the need for effective interdisciplinary communication and coordination. |
| Johnson, S. et al, 2011 [69] | Development and validation of a VR simulator for interventional radiology training, including two workstations simulating different steps of the Seldinger procedure for safe, realistic practice. | Performance on Seldinger procedure steps, realism of the simulation, usability, and transferability of skills to real-world procedures. | Construct validity confirmed; consultants outperformed trainees. Simulator-trained trainees performed better on real-world procedures | Some aspects lacked realism; technical issues with specific metrics; consultants may have taken the simulation less seriously than trainees. |
| Thoirs, K., et al., 2011 [70] | Simulated learning programs (SLPs) complementing traditional MRS education, including anatomical models, phantoms, computed radiography, image analysis, interactive group work, live actors, digital radiography, VR software, and simulated clinical settings. | Key variables measured include the effectiveness of simulated learning in skill acquisition, knowledge retention, user engagement, and the perceived value of simulation compared to clinical placements. | Positive feedback on simulation for safe, controlled learning, repetition, remedial training, and feedback. Enhanced psychomotor skills, understanding of radiotherapy, plan evaluation, and anatomy. Viewed as an enhancement, not a replacement, for clinical training. | Challenges included resistance from professional and accrediting bodies, resource constraints, the need for curriculum redevelopment, and ensuring student equity in access to simulation. |
| Sun, J., et al. 2017 [71] | AR training system for simulating radiographic procedures with a phantom, position tracking device, and visible light source for safe practice, compared with the VR system, ProjectionVR. | Learning effectiveness, student engagement, skill acquisition, ability to set SID, and capture radiographs. | AR system showed higher learning effectiveness due to more hands-on practice. Both AR and VR systems enhanced engagement and confidence with radiographic equipment. | AR system required further development of modules like collimation and filtration; subjective bias in observational assessments. |
| Gawlik-Kobylińska, M., et al. 2018 [72] | Digital filmmaking in VR for training CBRN first responders, involving live actions in a 3D environment and creating instructional videos using special software. | Key variables measured include awareness of CBRN threats, skill acquisition in handling hazardous situations, user engagement, and retention of learned procedures. | Digital filmmaking in VR for training CBRN first responders, involving live actions in a 3D environment and creating instructional videos using special software. | Potential barriers include the need for adequate digital skills among students and teachers, access to appropriate hardware and software, and individual preferences regarding learning in virtual environments |
| Süncksen, M, et al. 2018 [73] | Digital filmmaking in VR for training CBRN first responders, involving live actions in a 3D environment and creating instructional videos using special software. | Key variables measured include image accuracy, time taken to achieve the radiograph, overall radiation exposure, user engagement, and user satisfaction. | The intervention resulted in high user satisfaction and engagement, with users finding the system to be user-friendly and sufficiently realistic. The gamified approach motivated users and provided a high educational value for intraoperative C-arm imaging. | Some participants noted issues with the realism of C-arm handling and the quality of radiographic images. Minor usability issues for persons of small stature were also mentioned. |

**Table S2.** Stage of immersive technology considering compliance and limitations

| **Reference** | **Stage of Immersive** | **Type of IVT** | **Compliance** | **Limitations** |
| --- | --- | --- | --- | --- |
| Rowe, D., et al. 2023 [37] | Fully implemented | VR headset | High engagement with 188 volunteers participating in the study | Limited to first-year students at one university; further research needed for complex procedures and other settings |
| Shah et al., 2022 [38] | Pilot | Cardboard viewer VR (CVVR), Integrated headset VR (IHVR) | High compliance, majority completed last VR session close to the procedure | Single-institution study, small sample size, no investigation of 3D-video, limited scope to immersive video |
| Ryu et al., 2022 [39] | Fully implemented | Mixed reality using HoloLens2 glasses | Not specified | Single patient with lateral lymph node dissection outside study period |
| Pastor et al., 2022 [40] | Prototype | Digital simulation with optical tracking and simulated fluoroscopy | Not specified | Small number of participants, potential learning effect among novices, exclusion of task completion time |
| Chen et al., 2022 [41] | Prototype | VR headset (HTC VIVE Pro Eye), tracked tablet, Logitech VR Pen. | High, all participants completed tasks as instructed. | Small sample size, limited VR experience among participants, results may not generalize to all clinical settings. |
| Kiryukhin et al. 2022 [42] | Fully implemented | Virtual simulation | High engagement reported | Details on sample size and control group not provided |
| Bridge et al., 2021 [43] | Various stages including fully implemented | VR simulators, computer-based systems, simulated patients | Varied, increased uptake during COVID-19 | Small sample size, respondent bias, geographical limitations |
| Ma et al. 2011 [44] | Prototype | VR, Physical mannequin Monte Carlo simulation package | Not reported | Types of abnormalities that can be simulated Limits in simulating breathing and other motion artifacts |
| Wang et al., 2021 [45] | Fully implemented | VR simulator (TraumaVision) | High engagement, all participants completed the study | No significant improvement in radiation use or overall score |
| Gunn et al., 2021 [46] | Fully implemented | VR CT simulation | High engagement reported | Self-reported confidence, no pre/post knowledge skill test, changing course structure |
| Martin-Gomez et al., 2020 [47] | Proof-of-concept | AR headset (HoloLens2) | High engagement reported in preliminary study | Preliminary study, small sample size, need for comprehensive patient study |
| Taunk et al., 2021 [48] | Fully implemented | VR headset | High engagement reported | Small sample size, single institution study |
| Czaplinski et al, 2020 [49] | Fully implemented | VERT simulation, online modules, face-to-face tutorials | Increased engagement reported, varied responses over two years | Small sample size, low response rate, single institution study |
| Kazuki Nishi et al., 2020 [50] | Prototype | AR application | High engagement reported in preliminary study | Preliminary study, small sample size, need for comprehensive study |
| Kang et al., 2020 [51] | Prototype | AR tools (Microsoft HoloLens) | High engagement, positive feedback | Small sample size, single institution study |
| Johnson et al., 2020 [52] | Prototype | 360-degree VR video | High engagement, constructive feedback provided | Small sample size, single institution study, English-speaking only, potential researcher bias |
| Park et al., 2020 [53] | Prototype | AR headset (HoloLens) | High engagement in AR group | Small sample size, single-center study, potential implicit bias from using AR |
| Sapkaroski et al., 2019 [54] | Prototype | VR headset (Oculus Rift), Leap Motion controllers | High engagement in both groups, more self-paced learning in VR group | Small sample size, single institution study, limited to hand positioning training |
| Jones et al., 2019 [55] | Prototype | VR headset (Oculus Rift DK2), haptic device (3D Systems Touch) | High engagement reported by preliminary feedback from expert surgeons | Preliminary study, small sample size, lack of detailed participant demographics |
| Ja-Young et al., 2019 [56] | Prototype | AR application on a mobile device (e.g., Galaxy Note 4 or S8). | High engagement reported. | Preliminary study, small sample size, lack of detailed participant demographics, only available for iOS users initially. |
| Ryan, E. et al., 2019 [57] | Fully implemented | Virtual reality (VR) | 100% response rate for initial questionnaires, 62.5% response rate for retention questionnaire | Small sample size, potential bias from single researcher conducting both talks |
| Popovic, B, et al., 2019 [58] | Fully implemented | Virtual reality simulator (Simbionix Angio-Mentor) | High engagement with 100% compliance in virtual training and real-life practice | Small sample size, single center, potential biases, difficulty isolating simulation effects, subjective skills assessment. |
| Fernández, C., et al., 2018 [59] | Not explicitly mentioned | VR device similar to Google Cardboard | Not applicable | The study emphasizes the need for refined regulatory compliance testing that considers age-specific absorption rates and the limitations of current SAR testing methods |
| Guo, J., et al., 2018 [60] | Fully implemented | Augmented Reality (AR), smart gloves, virtual buttons | Higher engagement and interest due to interactive and visual nature of the AR system | System is currently more suitable for beginners and not as professional as traditional software, needs further development to increase functionality |
| Sugand, K. et al., 2018 [61] | Fully implemented | Augmented Reality (AR) | High compliance as all participants completed the task | Did not record the number of DHS procedures per participant, potential influence of time constraints, and lack of consideration for hand dominance. |
| Sapkaroski, D., et al., 2018 [62] | Fully implemented | Virtual Reality (VR) with haptic feedback and dynamic interaction capabilitie | High engagement with voluntary completion of the questionnaire | Comparison limited to consecutive year groups, based on perceived improvements, confined to hand positioning task. |
| Gunn, T., et al., 2017 [63] | Fully implemented | Virtual Reality (VR) simulation | High engagement with voluntary completion of the role-play assessments; 45 students' data were included in the final analysis. | Assessed only technical skill acquisition; did not evaluate student satisfaction or enjoyment. Further research needed for clinical outcomes and student confidence. |
| Chamunyonga, C. et al., 2017 [64] | Fully implemented | Virtual Reality (VR) system | High engagement in collaborative plan evaluation sessions | The study relies on anecdotal evidence and qualitative assessment; further quantitative research is needed to measure clinical performance and outcomes. |
| Diotte, B., et al., 2014 [65] | Prototype | Augmented Reality (AR) fluoroscope | High engagement and compliance, with all participants completing the trials | Conducted on dry bone phantoms; further research needed in clinical settings; small sample size limits generalizability. |
| Szőke, I., et al., 2014 [66] | Fully implemented | Virtual Reality (VR) simulation system | High engagement due to the practical application in real-world scenarios. | Primarily descriptive study focused on system implementation, lacking quantitative measurement of educational outcomes. |
| Nishi, K., et al. 2021 [67] | Fully implemented | Web-based Augmented Reality (WebAR) | High engagement due to the ease of access and interactive nature of the WebAR system, enabling users to observe radiation behavior from various angles. | Need for further development to enhance system performance and reduce data size. Recommended additional features to measure user comprehension and address usability differences across devices. |
| Freudenthal, A., et al, 2011. [68] | Prototype | Augmented Reality (AR), 3D navigation systems, robotic systems, haptic feedback. | High engagement from multidisciplinary teams, with active involvement in the design, development, and testing phases. | Complexity of integrating multiple technologies, need for iterative testing and development, and extensive interdisciplinary collaboration. |
| Johnson, S. et al, 2011 [69] | Prototype | Virtual Reality (VR) simulator | High engagement was observed, with participants completing simulation trials and providing feedback on the simulator's realism and usability. | Small sample sizes, especially in the transferability assessment phase; further development and validation needed to fully realize VR simulators' potential in medical training. |
| Thoirs, K., et al., 2011 [70] | Fully implemented | Virtual reality software, anatomical models, full body phantoms, computed radiography, image analysis software, digital radiography equipment. | High engagement was observed, with participants actively participating in interviews, surveys, and consultative meetings | Restricted by funding body objectives, excluding student perceptions; potential participant and researcher bias; limited evidence on SLPs' effectiveness in clinical skills development. |
| Sun, J., et al. 2017 [71] | Fully implemented | Augmented Reality (AR) and Virtual Reality (VR) | High engagement, with students actively participating in the learning activities and assessments. | Developmental stage of the AR system lacked some functionalities compared to state-of-the-art VR systems. Small sample size and need for further validation. |
| Gawlik-Kobylińska, M., et al. 2018 [72] | Fully implemented | Virtual Reality (VR) and digital filmmaking software | High engagement expected due to the interactive and immersive nature of the training | Conceptual study; further empirical research needed. Potential legal issues with digital content ownership and role blurring. |
| Süncksen, M, et al. 2018 [73] | Prototype | Virtual Reality (VR) headset, gamification software | High compliance and engagement, with users actively participating in the tasks and providing positive feedback on the VR system. | The study is primarily descriptive, focusing on user evaluations. Further empirical research is needed to validate the educational effectiveness and to address technical limitations related to C-arm handling and image quality. |

**Table S3.** Details about the implementation of the immersive environments

| **Reference** | **Number of Participants** | **Study Design** | **Duration of Intervention** | **Assessment Tools** | **Training/Instruction Provided** | **Context/Setting** | **Participant Demographics** | **Control/Comparison Group** |
| --- | --- | --- | --- | --- | --- | --- | --- | --- |
| Rowe, D., et al. 2023 [37] | 188 first-year radiography students (94 in VR group, 94 in physical simulation group) | Randomized controlled trial | 25 weeks | Objective Structured Clinical Examination (OSCE) using actors as patients in a physical X-ray environment | Both groups received training and familiarization. VR group used Virtual Medical Coaching’s software; physical group used Philips X-ray equipment. | University setting, involving both on-campus simulation laboratory and clinical departments for orientation. | First-year radiography students stratified by grade average and age | The study compared the VR simulation cohort with the physical simulation cohort |
| Shah et al., 2022 [38] | 13 | Randomized controlled trial | Minimum 6 weeks | Pre/post-tests, surveys, timed procedures, implant quality metrics | Unlimited access to VR technology, slideshows, 2D videos, and pelvic simulator | Radiation oncology residency program, clinical environment | 84.5% male, PGY-2 to PGY-5, mixed prior brachytherapy experience | Comparison between CVVR and IHVR |
| Ryu et al., 2022 [39] | 13 | Clinical study | Between August and September 2021 | Visibility assessments, surgical outcomes, NASA TLX questionnaire | 3D hologram creation from CT scans, usage of Holoeyes MD system | Surgical environment, colorectal cancer surgeries | 13 patients with colorectal cancer | Not reported |
| Pastor et al., 2022 [40] | 53 | Validation study with construct and face validity assessment | Not reported | Performance metrics, face validity questionnaire, Likert scale | Standardized introduction to the system and task | Training course at AO Davos Courses 2021 | Novices (29): orthopaedic trauma residents; Experts (24): consultants or chief surgeons | Comparison between novices and experts |
| Chen et al., 2022 [41] | 8 | Within-subject study. | Not reported | Pre-study questionnaire, post-study UX evaluations, DSC, task completion time, gaze analysis. | Introduction to VR and contouring, familiarization with the system before each session | Laboratory setting. | Mixed (2 females, 6 males, ages 24-27), all with human anatomy curriculum experience | Not reported |
| Kiryukhin et al. 2022 [42] | Not reported | Observational study | Not reported | Surveys, assessments of understanding and engagement | Guided practice sessions, interactive learning modules | Educational environment, nuclear physics training | Not reported | Not reported |
| Bridge et al., 2021 [43] | 72 responses from various institutions | International survey and audit | Not reported | Surveys, qualitative feedback, descriptive statistics | Guided simulations, interactive sessions, feedback mechanisms | Academic settings, clinical simulations | Varied, representing multiple countries and institutions | Not reported |
| Ma et al. 2011 [44] | 18 | Comparative study | Not reported | Performance data, participant ratings | Standardized task completion on simulator | Academic setting, simulation-based training | Urology residents, fellows, and faculty with varying levels of PCNL experience | PercMentor VR simulator, porcine tissue simulator |
| Wang et al., 2021 [45] | 28 | Randomized controlled trial | Not reported | Simulator-collected task completion time, success rate, radiation exposure time, overall score | Pretest, posttest, three additional simulator training sessions for the intervention group | Academic setting, orthopaedic surgery training | Medical and premedical students, novice trainees | Control group with no additional simulator training |
| Gunn et al., 2021 [46] | 66 (28 MI students, 38 RT students) | Survey study | Not reported | Surveys using a 5-point Likert scale | Instructor-guided tutorials, individual self-directed learning | Educational setting, undergraduate program at Queensland University of Technology | MI and RT undergraduate students, varied ages | Not reported |
| Martin-Gomez et al., 2020 [47] | Not reported, preliminary study with one team member | Proof-of-concept study | Not reported | Airflow rate consistency, user feedback | Pre-interventional session, user guidance during AR experience | Radiotherapy suite, academic setting | Not reported, preliminary study | No visual feedback, graph-based user interface |
| Taunk et al., 2021 [48] | 14 | Randomized controlled trial | Not reported | Pre/post-tests, timed procedures, implant quality metrics, questionnaires | VR training video, timed intracavitary procedure on pelvic simulator | Clinical and educational setting | 5 PGY-2, 3 PGY-3, 4 PGY-4, 2 PGY-5, 71.4% male, 35.7% with prior experience | No comparison group specified |
| Czaplinski et al, 2020 [49] | 24 in the first year, 11 in the second year | Survey study over two years | 13 weeks per year | Surveys, self-assessment quizzes, practical reports, final examinations | Online self-study, self-testing, group work, discussions, VERT simulations, clinical practicals | Educational setting, Queensland University of Technology | Medical physics students, diverse backgrounds | Not reported |
| Kazuki Nishi et al., 2020 [50] | Not reported, preliminary study | Prototype development and preliminary testing | Not reported | Monte Carlo simulations, physical measurements, user feedback | Guided use of AR application | Educational and clinical setting | Not reported, preliminary study | Not reported |
| Kang et al., 2020 [51] | 32 participants (19 medical physics students, 2 medical physics residents, 11 radiation oncology residents) | Mixed-methods pilot study | Two 2-hour sessions each semester for two semesters | Surveys with Likert scale and short-answer questions, qualitative analysis | Didactic lectures, pre-lab contouring assignments, AR-enhanced lab sessions | Educational setting, McGill University | Medical physics and radiation oncology students and residents, age range 21-36 | Not reported |
| Johnson et al., 2020 [52] | 7 participants | Pilot study using focus groups | Not reported | Thematic analysis of focus group discussions | Traditional first day patient teaching session, VR video viewing during focus group | Single academic cancer centre, focus group setting | Patients receiving image-guided EBRT to the pelvis, ages 54-67, mix of male and female | Traditional verbal and/or written education |
| Park et al., 2020 [53] | 12 in AR group, 16 in prospective control group, 15 in retrospective control group | Prospective control study with retrospective controls added for statistical power | Not reported | Fluoroscopy time, catheterization time, dose-area product (DAP), air kerma (AK) measurements | Preprocedural MR imaging, AR visualization setup | Preclinical setting with rats as subjects | Rats with diethylnitrosamine-induced hepatocellular carcinoma, average weight around 446 grams | Prospective control group, retrospective control group |
| Sapkaroski et al., 2019 [54] | 76 students (19 males, 57 females) | Split-cohort study | Not reported | 5-point Likert scale questionnaire, thematic analysis of qualitative feedback | Hands-on tutorial for VR group, instructional booklet for role-play group | Educational setting, Monash University | Year 1 undergraduate radiography students, average age 20.4 ± 1.8 years | Traditional clinical role-play group |
| Jones et al., 2019 [55] | Not reported in the provided excerpt | Quantitative comparison study with qualitative feedback | Not reported in the provided excerpt | Performance metrics tracking, visual comparison with fluoroscopic images, paired t-tests for deformation angles | Hands-on tutorial for using the VR system and haptic device | Preclinical setting, university laboratory | Not reported in the provided excerpt | Fluoroscopic images of actual electrode insertion for comparison |
| Ja-Young et al., 2019 [56] | Not reported in the provided excerpt. | Descriptive study on the development and application of the AR simulator | Not reported in the provided excerpt. | Real-time feedback, user engagement tracking, qualitative feedback. | Instructions for using the AR application and performing radiographic techniques. | Educational setting, used for preclinical training in dental schools. | Dental hygiene students in preclinical training stages. | Not reported. |
| Ryan, E. et al., 2019 [57] | 40 | Randomized controlled trial | Not explicitly mentioned (Two sessions were held) | Likert scale questionnaires, multiple-choice questions, open-ended questions | Detailed teaching sessions on radiotherapy setup, verification, and protocols using VLE | Academic environment (Trinity College Dublin, School of Medicine) | Undergraduate students from various disciplines except radiotherapy | Traditional didactic lecture |
| Popovic, B, et al., 2019 [58] | 20 cardiology residents | Randomized controlled trial | Not explicitly mentioned (training sessions were held) | rocedural flow checklist, performance score, clinical parameters, and statistical analysis | Didactic teaching, virtual training sessions, real-life practice under supervision | Catheterization laboratory and virtual simulation environment | Cardiology residents, mean age approximately 26-27 years, mixed gender | Traditional catheterization laboratory mentor-based training |
| Fernández, C., et al., 2018 [59] | Not applicable (study based on simulations and models) | Simulation study using anatomical models and computational methods | Not applicable (simulation-based study) | Finite-difference time-domain (FDTD) simulation, SAR measurements in 1 g and 10 g cubes of tissue | Not applicable | Virtual environment simulation | Models representing a range of ages from 3 to 34 years, including specific models for children and adults | Comparison between child and adult models in terms of SAR and radiation absorption |
| Guo, J., et al., 2018 [60] | Not reported (focus on system capabilities and educational impact rather than a specific participant study) | Not explicitly mentioned (descriptive study of the AR system's development and capabilities) | Not reported (system design and application description) | Not reported (focus on system description and potential educational benefits) | Description of how to use the AR system with smart gloves and virtual buttons | Educational environment, specifically for teaching antenna design | Not reported (general focus on students and beginners in antenna design) | Comparison made with traditional antenna design software and other educational methods (not a specific control group but a comparative analysis) |
| Sugand, K. et al., 2018 [61] | 26 surgeons | Multicenter study with a validation design | Each participant completed one attempt at the simulation task | Real-time performance metrics and a 7-point Likert scale for face and content validity. | Standardized explanation of the task and objective metrics to participants before the simulation attempt | Orthopedic training environments in multiple hospitals (Northwick Park, Central Middlesex, Princess Alexandra Hospital) | Surgeons categorized into novice (<10 DHS procedures), intermediate (10-39 DHS procedures), and expert (≥40 DHS procedures) groups | Comparison between novice, intermediate, and expert surgeons |
| Sapkaroski, D., et al., 2018 [62] | 79 first-year radiography students in 2016 | Comparative study using a 5-point Likert scale questionnaire to assess student perceptions | 45-minute sessions as part of a 3-hour laboratory class | 5-point Likert scale questionnaire | Students were instructed to complete an integrated hand-imaging lesson plan and associated imaging referral for a PA and lateral hand, involving patient communication, positioning, and selection of imaging parameters. | Classroom/laboratory environment at Monash University | First-year radiography students | Comparison with the 2015 cohort using Shaderware simulation tool |
| Gunn, T., et al., 2017 [63] | 57 students initially, with 45 students' data suitable for direct comparison | Comparative study with experimental design | The exact duration of each VR session is Not reported, but the study involved repeated role-play assessments. | Role-play assessments using a detailed rubric for technical skill breakdown and marking guide, with statistical analysis of role-play scores | Didactic lectures on medical imaging positioning protocols before practical practice in VR simulation or traditional laboratory. | Classroom and laboratory setting at Queensland University of Technology (QUT) | First-year undergraduate medical imaging students, with demographic factors such as age, gender, and gaming skills recorded | Comparison between students using VR simulation and those using traditional laboratory simulation |
| Chamunyonga, C. et al., 2017 [64] | Not reported in detail | Descriptive and qualitative study | Not explicitly mentioned | Qualitative plan evaluation using visual analysis of isodose distribution, VERT system features for dose coverage and plan delivery visualization | Collaborative treatment plan evaluation sessions, export and visualization of treatment plans on VERT | Educational environment at Queensland University of Technology (QUT) | Undergraduate radiation therapy students | Comparison with traditional teaching methods not explicitly mentioned, focus on VERT's educational impact |
| Diotte, B., et al., 2014 [65] | 6 surgeons | Preclinical study involving dry bone phantoms | Not explicitly mentioned (single session involving multiple trials) | Success/fail metrics for the procedure, number of X-ray images, navigation time, and feedback from participating surgeons | Surgeons were instructed to perform the distal locking procedure using the AR fluoroscope system on dry bone phantoms | Preclinical setting using dry bone phantoms in a simulated surgical environment | Surgeons with varying levels of expertise (experts, residents, novices) | Not explicitly mentioned (focus on the efficacy of the AR system) |
| Szőke, I., et al., 2014 [66] | The study does not specify the exact number of participants but mentions extensive use in real-world activities. | Descriptive study with a focus on the development and implementation of the VRdose and Halden Planner systems | Not explicitly mentioned, as it covers various applications over an extended period. | Radiation risk assessment techniques, visualization of radiation fields, and calculation of radiation dose using 3D simulation tools. | Training included the use of the VRdose system for visualizing radiation fields and calculating dose, supporting work planning, and reviewing current work practices. | The intervention was set in nuclear environments for supporting work simulation and radiation risk assessment. | Not reported in detail, but implies involvement of nuclear industry workers and trainees. | The study does not mention a specific control or comparison group. |
| Nishi, K., et al. 2021 [67] | The specific number of participants involved in the initial evaluations is not mentioned. | Descriptive study focusing on the development, implementation, and initial testing of the WebAR system for radiation protection education. | Not explicitly mentioned; involved development, testing, and user feedback sessions. | Observational assessments, user feedback on system usability, and operational tests on multiple devices. | Users were provided with access to the WebAR system via a URL or QR code, enabling them to use their device's camera to observe the 3D visualization of scattered radiation. | Educational settings for radiation protection training, suitable for use in classrooms, laboratories, and clinical environments. | Not reported in detail; generally aimed at radiological workers and students in medical physics and radiation protection fields. | The study compares the WebAR system with conventional AR/VR systems that require special devices or application downloads. |
| Freudenthal, A., et al, 2011. [68] | Collaborative effort involving engineers, Human Factors specialists, industrial designers, and medical end-users. | Descriptive study focusing on collaborative co-design and the integration of emerging technologies for surgical applications. | The duration is ongoing, as it includes iterative development and testing phases over several years. | User feedback, heuristic evaluations, workflow integration matrices, observational studies, and prototype testing. | Robotics courses, laparoscopic training programs, user-centered design courses, hands-on workshops, and real-world scenario testing. | The setting includes academic institutions, partner hospitals, and industrial partners, focusing on both educational and practical applications in surgical environments. | Participants include PhD students, postdoctoral researchers, engineers, Human Factors specialists, industrial designers, and medical end-users, with a focus on multidisciplinary team collaboration. | Not explicitly mentioned, but the study compares current surgical practices with the new ARIS*ER system. |
| Johnson, S. et al, 2011 [69] | Study 1: 35 consultant interventional radiologists.  Study 2: 62 participants (trainees and consultants).  Study 3: 14 IR trainees for transferability assessment. | Phase 1: Cognitive task analysis to inform simulator design.  Phase 2: Validation for face and construct validity.  Phase 3: Assessment of skill transferability from simulator to real-world procedures. | Not explicitly mentioned; varied depending on study phase. | Performance metrics on the simulator, background questionnaires, procedure-based assessment forms for real-world procedures. | Participants received written instructions, information sheets on the Seldinger procedure, and verbal instructions from experimenters. Simulator training sessions included performing trials on different patient cases and receiving performance feedback. | The study was conducted across multiple hospitals and conferences, with settings including academic institutions and clinical environments. | Participants included consultant interventional radiologists, vascular surgeons, and IR trainees with varying levels of experience. | The study included a control group of IR trainees who did not receive simulator training for the transferability assessment phase. |
| Thoirs, K., et al., 2011 [70] | 205 participants, including educators, professional associations, accrediting bodies, and clinicians across MRS disciplines. | Descriptive study using semi-structured telephone interviews, online surveys, and face-to-face consultative meetings to gather data on the use and perceptions of simulation in MRS education. | The study was conducted from September to November 2010. | Semi-structured interviews, online surveys, face-to-face meetings, literature reviews. | Training included the use of SLPs in various formats, such as virtual radiography systems, avatars for clinical scenarios, and the Virtual Environment for Radiotherapy Training (VERT) system. | Educational environments at Australian universities, private sonography schools, and international MRS programs. | Participants included Australian academics, educators from private sonography schools, international MRS educators, representatives from professional and accrediting bodies, and clinicians across the four MRS disciplines. | The study did not have a specific control group but compared traditional clinical training methods with simulated learning. |
| Sun, J., et al. 2017 [71] | 10 groups of year 2 medical imaging students, with each group consisting of 3 students, totaling 30 participants. | Comparative study using both AR and VR systems, with a formative assessment approach to measure learning outcomes. | The lab session lasted 3 hours. | Formative assessments with questionnaires based on Donald Kirkpatrick’s four-level evaluation model and practical tasks for capturing radiographs with specified SIDs. | Students received instructional materials and time to familiarize with AR and VR systems, practiced radiographic techniques, and completed performance-based assessments. | Educational setting at Shanghai University of Medicine and Health Sciences. | Year 2 medical imaging students with basic lecture knowledge of radiography. | Comparison between AR and VR systems |
| Gawlik-Kobylińska, M., et al. 2018 [72] | Not explicitly mentioned | Descriptive study presenting the concept and potential applications of digital filmmaking in virtual reality for CBRN training | Not explicitly mentioned | Qualitative assessments through observations and feedback on the effectiveness of the training and instructional videos | Training includes performing live actions in virtual reality scenarios, recording these actions, and creating instructional videos. Students receive guidance on creating high-quality instructional materials and are evaluated on their ability to create effective training videos. | Educational and training settings for CBRN first responders | Not reported in detail, but includes CBRN first responders and students undergoing preparatory training | Not explicitly mentioned |
| Süncksen, M, et al. 2018 [73] | Initial evaluation: 9 non-medical users  Second evaluation: 68 healthcare professionals (operating room personnel), with 41 providing written feedback | Descriptive study with user evaluations through feedback forms and the User Experience Questionnaire (UEQ) | Sessions lasted approximately one hour each for initial evaluation; the duration for the second evaluation is Not reported but involved brief training sessions during a course. | User Experience Questionnaire (UEQ), feedback forms, and observational assessments during VR interactions | Users received brief introductions to the utilization of mobile image intensifiers in the operating room, followed by hands-on practice with the VR system to take radiographs and solve tasks. | Educational and training settings, including initial evaluations with non-medical users and further evaluations with healthcare professionals in a training course. | Initial evaluation: 9 non-medical users  Second evaluation: 41 healthcare professionals with varying levels of experience with mobile X-ray devices (mean experience of 8.7 years) | No specific control group mentioned; comparisons are made between desktop and VR modes within the study. |
